# Dynamic BMP gene expression regulation in chick RPE during recovery from short term optical defocus and form-deprivation

**DOI:** 10.1371/journal.pone.0311505

**Published:** 2024-10-11

**Authors:** Yan Zhang, Qiurong Zhu, Wulian Song, Grace May Chuang, Daniel Sun, Kiana Cheung, Andreana Chou, Andrea He, Elham Shoghi, Christine F. Wildsoet

**Affiliations:** 1 Herbert Wertheim School Optometry and Vision Science, University of California, Berkeley, California, United States of America; 2 Department of Ophthalmology, Schepens Eye Research Institute of Massachusetts Eye and Ear, Harvard Medical School, Boston, Massachusetts, United States of America; 3 Department of Ophthalmology, the 2nd Affiliated Hospital, Harbin Medical University, Harbin, Heilongjiang, China; Saarland University, GERMANY

## Abstract

**Purpose:**

This study investigated the differential gene expression of BMPs in chick retinal pigment epithelium (RPE) during recovery from short term exposure to optical defocus and form-deprivation (FD) treatments.

**Methods:**

14-day old White-Leghorn chicks wore either monocular +10 or -10 D lenses, or diffusers for 2 or 48 h, after which eyes were allowed unobstructed vision for up to 96 h. Over this recovery period, refractive errors and choroidal thickness (ChT) were tracked using retinoscopy and high-frequency A-scan ultrasonography. Real-time PCR was used to examine the expression of BMP2, 4, and 7 genes in RPE samples collected 0, 15 min, 2, 24, 48, and 96 h after the termination of treatments. Expression levels in treated eyes and their contralateral control eyes were compared.

**Results:**

After the termination of the lens and diffuser treatments, eyes gradually recovered from induced shifts in refractive error. With all three treatments, ChT changes reached statistical significance after 48 h of treatment, be it thinning with the -10 D lens and diffuser treatments (-0.06 ± 0.03mm, *p* < 0.05; -0.11 ± 0.04 mm, *p* < 0.05, resp.), or thickening with the +10 D lens (0.31 ± 0.04 mm, *p* < 0.001). BMP2 gene expression was rapidly upregulated in eyes wearing the +10 D lens, being statistical significance after 2 h, as well as 48 h of treatment. With the 2 h treatment, the latter gene expression pattern persisted for 15 min into the recovery period, before decreasing to the same level as that of contralateral control eyes, with a short-lived rebound, i.e., upregulation, 24 h into the recovery period. With the longer, 48 h treatment, BMP2 gene expression decreased more gradually, from 739 ± 121% at the end of the treatment period, to 72 ± 14% after 48 h of recovery. Two and 48 h of both -10 D and FD treatments resulted in BMP2 gene expression downregulation, with the time taken for gene expression levels to fully recover varying with the duration of initial treatments. In both cases, BMP2 gene expression downregulation persisted for 15 min into the recovery period, but reversed to upregulation by 2 h. Similar gene expression patterns were also observed for BMP4, although the changes were smaller.

**Conclusions:**

The observed changes in BMP gene expression in chick RPE imply dynamic, albeit complex regulation, with the duration of exposure and recovery being critical variables for all three types of visual manipulations. This study provides further evidence for a role of the RPE as an important signal relay linking the retina to the choroid and sclera in eye growth regulation.

## Introduction

Myopia (near-sightedness) is the most common type of refractive error and is also one of the world’s leading causes of visual impairment and blindness [1]. In recent years, myopia has become recognized as a significant public health issue worldwide, with the prevalence of myopia already at epidemic levels in some Asian countries and continuing to rise worldwide [2–4]. When uncorrected, myopia results in blurred distant vision, a by-product of the relative increase in axial length compared to the eye’s optical (refracting) power [5]. While such mismatching errors can be corrected with optical aides, including spectacles and contact lenses, or refractive surgery, to restore clear vision, on the other hand, myopia is associated with increased risks of visual impairments tied to a variety of pathologies, including glaucoma, myopic maculopathy, retinal detachment, and cataracts [6]. While clinical treatment strategies to prevent and/or slow the progression of myopia are under investigation, with some multifocal contact lens options already in use [7], improved understanding of the underlying disease process is key to improving treatment efficacy, potentially via the development of novel therapies, including gene-based ones.

The important influences of visual experience, including optical defocus, on eye growth regulation has been demonstrated through both animal model studies, and studies in humans [8–15]. In the case of optical defocus, the rate of eye growth is adjusted in compensation, in a process known as emmetropization. For example, a negative defocusing lens, which imposes hyperopic defocus when placed over a normal, nearly emmetropic eye, accelerates eye elongation and the choroid thins, which together appropriately adjust the retina’s location to match the altered plane of focus [8, 16]. The opposite is true for a positive lens, which imposes myopic defocus and triggers thickening of the choroid and slowed eye elongation [16, 17]. Degrading retinal image contrast, for example by covering an eye with a diffuser, also induces myopia; such form-deprivation (FD) conditions accelerate eye elongation and thin the choroid, as do negative lenses, although here the imposed conditions are open loop [16]. When the inducing treatment is terminated, i.e., either lenses or diffusers are removed, these eyes initially exhibit refractive errors that reflect their altered choroidal thickness and eye lengths, hyperopia in the case of eyes that are shorter than normal with thickened choroids, and myopia, in the case of eyes that are longer than normal with thinned choroids. These induced refractive errors reactivate emmetropization, at least in young animals, allowing recovery from the same, although whether these “recovery responses” are mediated by same or different signaling pathways as activated by imposed optical defocus on emmetropic eyes remains under debate [18–20].

Although the etiology of human myopia is not yet well understood, animal studies have provided convincing evidence for local (ocular) growth regulatory mechanisms. For example, myopia may be induced using one of the above experimental manipulations, even in eyes that are disconnected from the brain by severing the optic nerve [16, 21]. These observations have been interpreted as evidence for a retina-to-sclera signaling cascade, in which detected changes in optical defocus and/or spatial contrast generates retinal signals that activate downstream signaling cascades targeting the outer layers of the eye wall, i.e., the choroid and sclera [8]. Being located between the retina and the choroid, the retinal pigment epithelium (RPE) is known to have important roles in the transportation of ions and fluids between the retina and choroid, as is critical for maintaining the functional integrity of the former tissue [22]. However, its strategic location, between the retina and the choroid, opens up the possibility that the RPE may also serve as a relay for retinal growth-regulatory signals directed at the choroid and sclera, with the net effects being either acceleration or slowing of the rate of elongation of the vitreous chamber through structural and/or dimensional changes in these tissues [23, 24].

Bone Morphogenetic Proteins (BMPs) were first discovered through their involvement in bone formation and osteogenesis, but have since been shown to have a broad range of important biological functions [25–27]. In the context of ocular growth regulation, our studies utilizing young chicks as a model, have documented significant, bidirectional changes in RPE gene expression for three BMPs, i.e., BMP2, 4, and 7, in response to imposed short-term, optical defocus of opposite sign [28–31]. Specifically, myopic defocus, which slows ocular elongation, led to rapid upregulation of BMP gene expression in RPE. Conversely, hyperopic defocus, which accelerates ocular elongation, led to rapid downregulation of BMP gene expression in RPE, as did FD. Thus overall, downregulation of BMP expression in RPE appears to be associated with accelerated ocular elongation, while upregulation of BMP expression is associated with slowed ocular elongation.

As noted above, when treatments used to experimentally induced refractive errors are terminated, the eyes of young animals are able to at least partly recover from induced changes. In the study reported here, which also made use of the chick as a model, we examined how differential BMP gene expression patterns change after optical defocus and FD treatments are terminated, thereby triggering recovery, and their temporal relationship with ocular biometric changes.

## Materials and methods

### Animals and visual treatments

White-Leghorn chicks were hatched from eggs supplied by University of California, Davis (Davis, CA), and raised in an animal facility at the University of California, Berkeley (Berkeley, CA), under 12 h/12 h light/dark cycle, with free access to food and water. Experiments were conducted according to the ARVO Statement for the Use of Animals in Ophthalmic and Vision Research, and approved by the Animal Care and Use Committee (ACUC) at University of California, Berkeley, CA.

**Fig 1:**
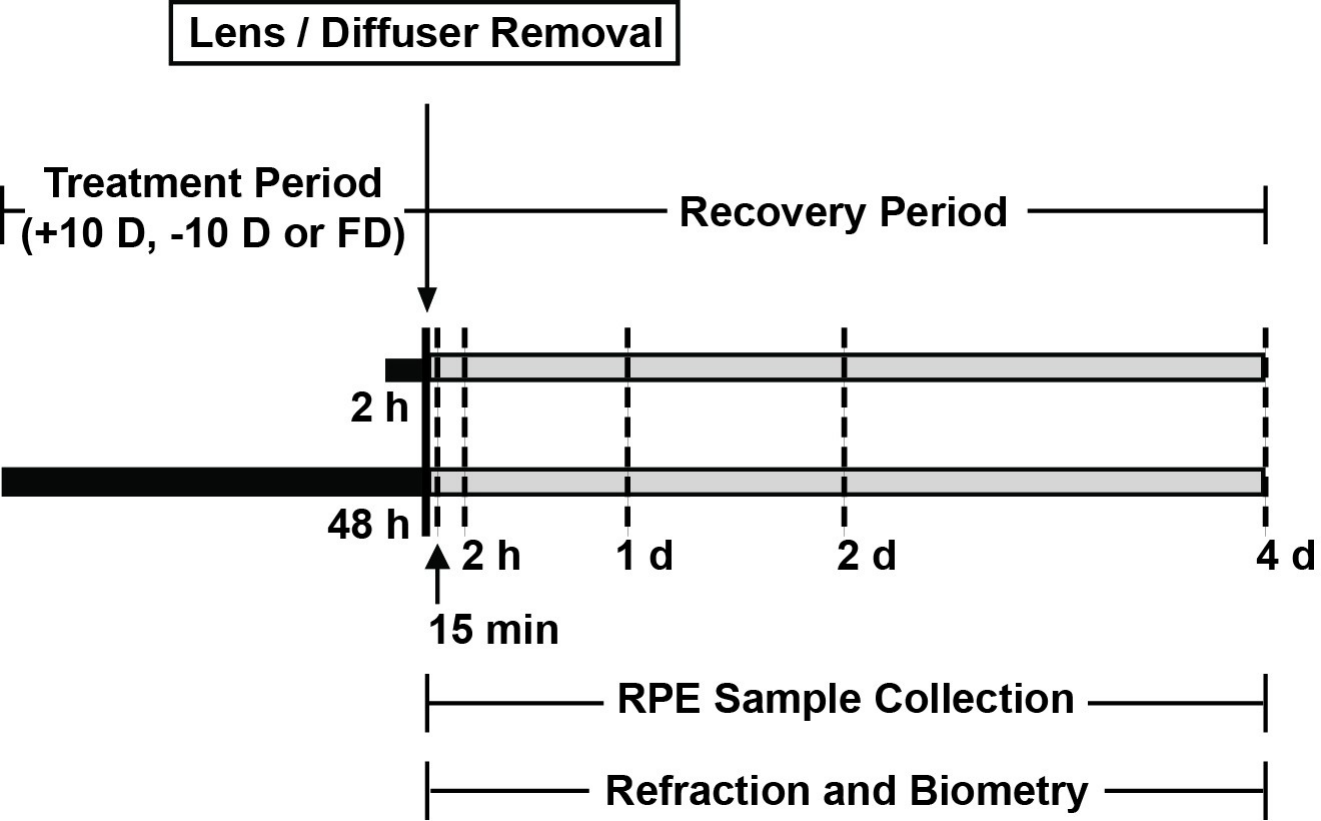
Diagram summarizing timing of lens/diffuser treatments (black bar), and recovery (gray bar) periods, as well as of *in vivo* ocular measurement and RPE sample collection.

At 14 days of age, chicks were fitted with either a +10 or -10 D spectacle lens or an opaque white diffuser over one eye, all Velcro-mounted, with contralateral fellow eyes left untreated as a control. Treatments were terminated after either 2 or 48 h, and eyes then monitored with timed *in vivo* ocular measurements for up to 96 h, ending at the time of RPE sample collection. Schedules for treatments, ocular measurements and sample collection are summarized in Fig 1. Groups ranged from 8 to 17 in size; specific numbers are as listed in the result sections.

### Refractive error and ocular biometric (choroidal thickness) measurements

Refractive errors and ocular dimensions were measured using retinoscopy and high-frequency A-scan ultrasonography respectively, both under gaseous anaesthesia (1.5% isoflurane in oxygen) [19]. Measurements were undertaken at the beginning of the recovery period (i.e., after either 2 or 48 h of treatment), and again at 2, 48 and 96 h into the recovery periods (Fig 1), with all measurements made in the afternoon to minimize the influence of diurnal rhythms.

### RPE isolation and RNA extraction

RPE samples were collected from both eyes of treated birds (treated and contralateral fellow eyes), at either the end of a 2 or 48 h treatment period, or 15 min, 2, 24, 48, 96 h into the recovery period (Fig 1). All samples were collected in the afternoon between 12–3 pm. In brief and as described in detail previously [28–31], chicks were sacrificed and their eyes immediately enucleated, after which the anterior segments of eyes were cut away at the equator to isolate the posterior segments. Next, the retina was removed to expose the RPE, which was then collected by gentle pipetting with cold PBS and subsequently lysed with RLT lysis buffer (RNeasy Mini kits, Qiagen, Valencia, CA), homogenized, and stored at -80° C for later use. Total RNA from RPE samples was purified using RNeasy Mini Kits (Qiagen), followed by on-column DNase digestion (Qiagen), according to the manufacturer’s protocol.

### BMP gene expression level measurement

After purification, RPE RNA was reverse transcribed to cDNA (SuperScript III First-Strand Synthesis System for RT-PCR, Invitrogen, Carlsbad, CA). The design of BMP2, 4, 7 primers and their validation have been described in previous studies [28, 29]. Gene expression levels were measured for BMP2, 4, and 7 by real-time PCR using iTaq Universal SYBR Green Supermix (Bio-Rad) and a StepOnePlus Real-Time PCR System (Life Technologies, Grand Island, NY). Glyceraldehyde-3-phosphate dehydrogenase (*GAPDH*) was used as the reference gene. All real-time PCR measurements were performed in triplicates. mRNA expression levels of target genes are represented as percentage (%) of treated versus fellow (control) eyes, with data for each treatment condition representing group averages.

### Statistical analysis

The data are presented as means and standard errors of the mean (SEM). One-way ANOVAs combined with post-hoc analysis (with Bonferroni correction) were used, with repeated measures ANOVAs used to examine temporal changes in interocular differences in refractive error and ocular biometric dimensions. Paired Student’s *t*-tests were used to compare gene expression levels in treated and fellow eyes, for each treatment condition.

## Results

### Refractive Error (RE) and Choroid Thickness (ChT) changes

**Fig 2:**
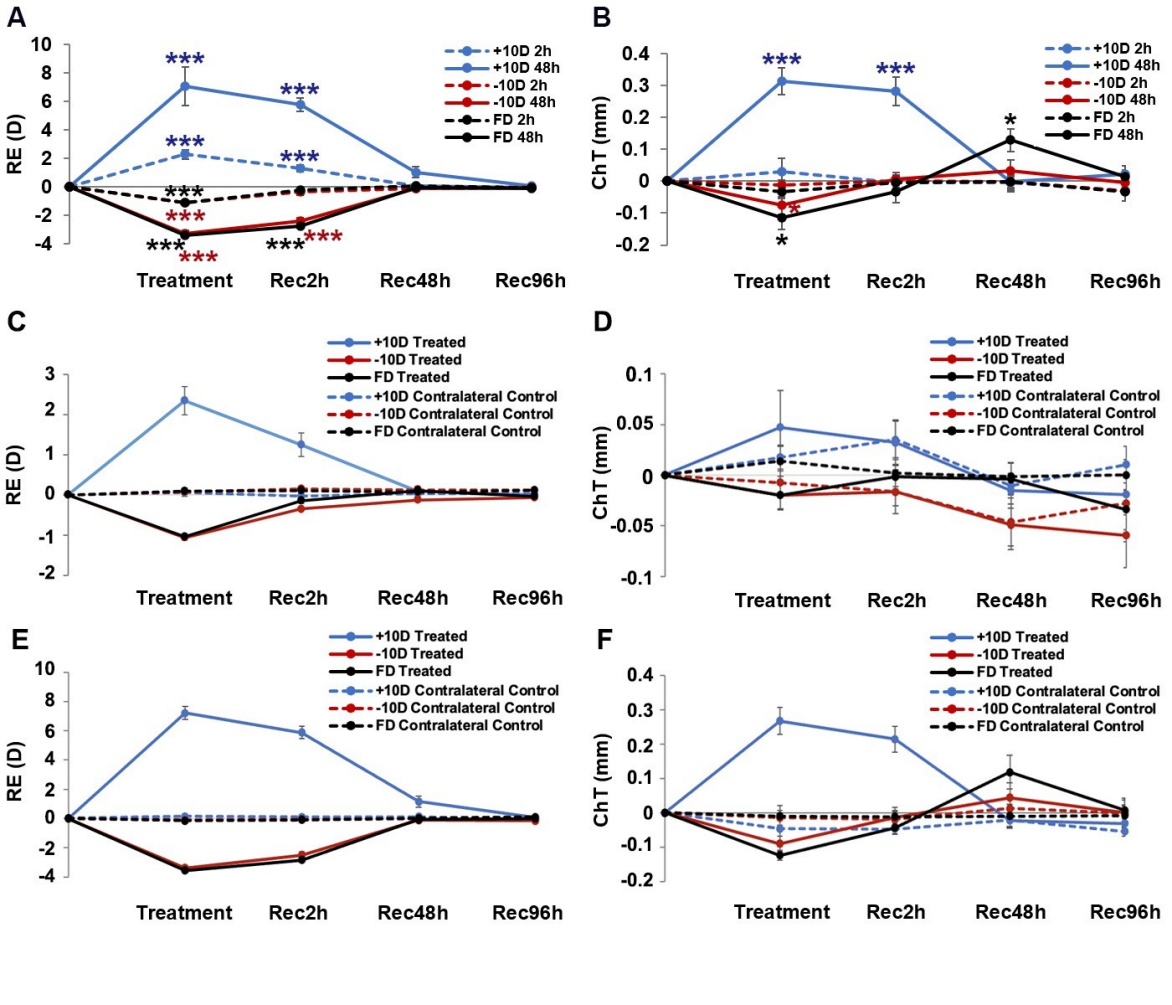
Effects of monocular +10 & -10 D lenses and diffuser (FD) treatments on refractive errors (RE, A, C, E) and choroidal thickness (ChT, B, D, F). Changes after 2 h or 48 h of treatment and up to 96 h of recovery (Rec), i.e., post treatment shown as interocular differences (treated-control eyes, mean ± SEM) are shown in A & B. Effects of these visual manipulations and recovery from the same on treated and contralateral fellow eyes are shown for 2h treatment, in C&D, and for 48 h treatment, in E &F. * *p* < 0.05, *** *p* < 0.001.

In relation to induced refractive errors, temporal profiles varied with both the type and duration of the inducing treatment, as well as recovery durations (Fig 2A, Table 1 and S1 File). Thus the +10 D lens induced significant hyperopic shifts in treated eyes relative to their fellows, after just 2 h of lens wear (+2.28 ± 0.31 D, n = 8, *p* < 0.001), albeit smaller than the change recorded after 48 h of wear (+7.05 ± 0.43 D, n = 10, *p* < 0.001). For both the 2 and 48 h treatment groups, these interocular differences decreased with time over the recovery period, with interocular differences being insignificant by 48 h into the recovery period. In contrast, the -10 D lens and diffuser treatments induced myopic shifts in treated eyes relative to their fellows, with interocular differences for the 2 and 48 h treatment durations being similar in magnitude for each of the two treatments (*p* < 0.001 for all cases). For both 2 h treatment groups (i.e., -10 D lens & diffuser), interocular differences in refractive error decreased rapidly after termination of the treatments, with interocular differences being insignficant just 2 h into the recovery period. While recovery was slower for both 48 h treatment groups, interocular differences were no longer significant by 48 h into the recovery period.

**Table 1:** Changes in interocular differences in refractive errors (RE) and choroidal thickness (ChT) induced by 2 or 48 h monocular treatment with either +10 D or -10 D lenses, or form depriving diffusers, and after recovery periods of up to 96 h.

	+10 D		- 10 D		Diffuser	
	RE (D)	ChT (mm)	RE (D)	ChT (mm)	RE (D)	ChT (mm)
**2 h Treatment duration**						
**0**	+2.28 ± 0.31***	0.03 ± 0.04	-1.13 ± 0.08***	-0.01 ± 0.02	-1.11 ± 0.14***	-0.03 ± 0.02
**2 h**	+1.28 ± 0.24***	-0.005 ± 0.02	-0.34 ± 0.17	-0.0001 ± 0.03	-0.21 ± 0.13	-0.004 ± 0.02
**48 h**	+0.06 ± 0.11	-0.005 ± 0.02	-0.13 ± 0.11	-0.003 ± 0.03	+0.07 ± 0.07	-0.003 ± 0.02
**96 h**	-0.03 ± 0.10	-0.03 ± 0.01	-0.03 ± 0.11	-0.03 ± 0.03	-0.07 ± 0.11	-0.03 ± 0.03
**48 h Treatment Recovery**						
**0**	+7.05 ± 0.43***	0.31 ± 0.04***	-3.29 ± 0.21***	-0.06 ± 0.03*	-3.41 ± 0.13***	-0.11 ± 0.04*
**2 h**	+5.78 ± 0.45***	0.28 ± 0.04***	-2.43 ± 0.24***	0.0003 ± 0.01	-2.75 ± 0.09***	-0.03 ± 0.03
**48 h**	+1.03 ± 0.38	-0.001 ± 0.02	-0.11 ± 0.07	0.02 ± 0.03	-0.06 ± 0.08	0.13 ± 0.04*
**96 h**	+0.03 ± 0.09	0.02 ± 0.01	-0.04 ± 0.12	-0.005 ± 0.03	-0.09 ± 0.07	0.01 ± 0.03

Data expressed as changes from baseline interocular differences (mean ± SEM).

* *p* < 0.05

*** *p* < 0.001.

In relation to choroidal thickness (ChT), the temporal profiles of induced changes also varied with both the type and duration of the inducing treatment, as well as the recovery duration (Fig 2B). As expected, the +10 D lens induced significant thickening, achieving statistical significance after 48 h of treatment (0.31 ± 0.04 mm, *p* < 0.001). The choroids of these eyes also remained significantly thicker than their fellows 2 h into the recovery period (0.28 ± 0.04 mm, *p* < 0.001), although were no longer different from the choroids of their fellows after 48 h of recovery. Also as expected, choroidal thinning was observed with both -10 D lens and diffuser treatments, although interocular differences reached statistical significance only with the longer, 48 h treatment (-10 D lens: -0.06 ± 0.03 mm, *p* < 0.05; diffuser: -0.11 ± 0.04 mm, *p* < 0.05). Over the recovery period after the latter treatments, the choroids of treated eyes rapidly thickened towards normal values, i.e., of fellow eyes and thus interocular differences rapidly decreased, to become statisitically insignificant over the 96 h recovery period after termination of the -10 D lens treatment. On the other hand, the recovery pattern for FD treatment group included a transient overshoot, with the choroids recording near normal values at the 2 h time point, before becoming transiently thicker than those of their fellows at the 48 h time point (0.13 ± 0.04 mm, *p* < 0.05).

**Fig 3:**
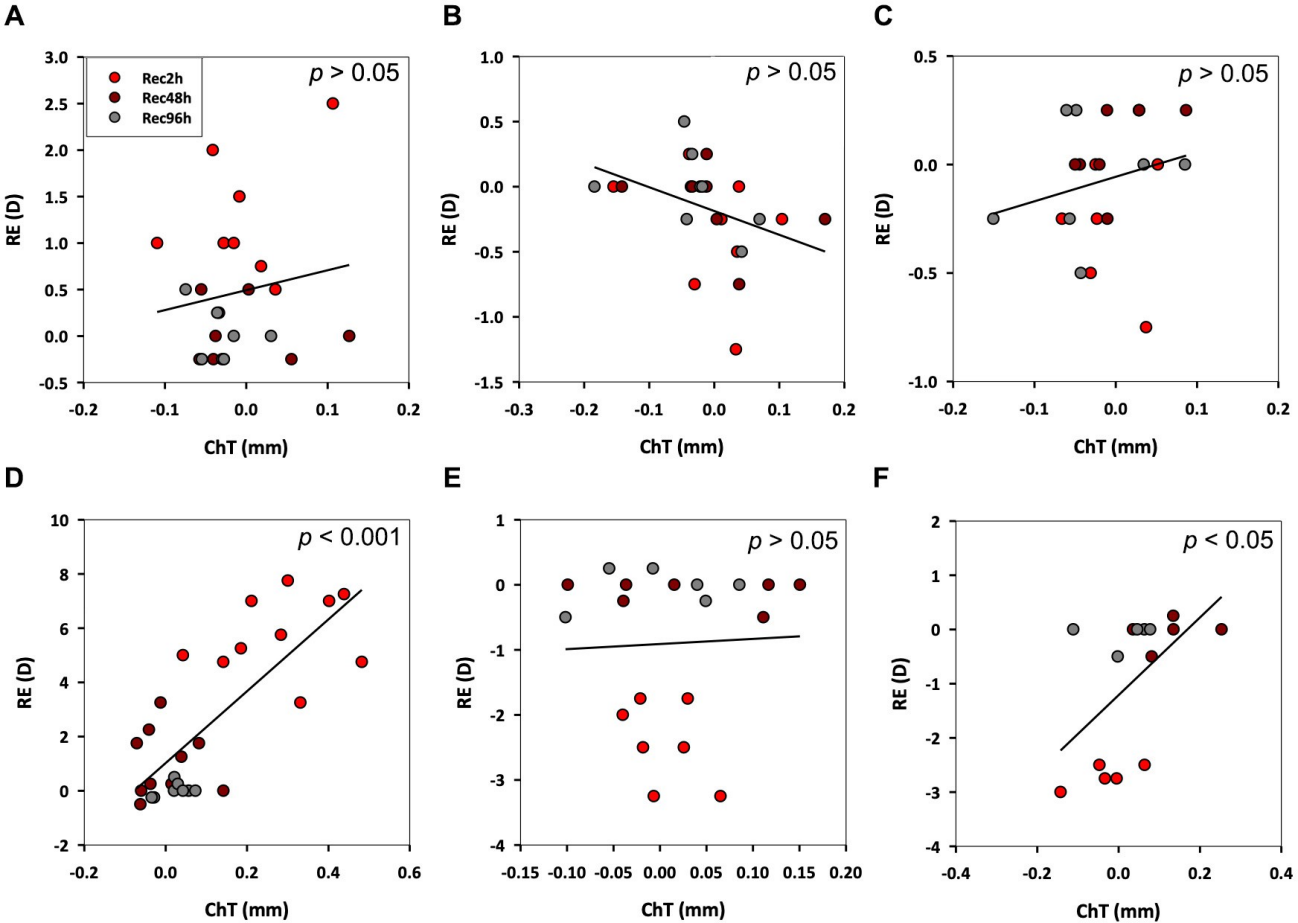
The relationships between change in RE vs. ChT for recovery from +10 D (A, D), -10 D (B, E), and FD (C, F) treatments, either 2 h (A, B, C) or 48 h (D, E, F), followed by up to 96 h of recovery.

The contribution of ChT changes to the recovery from REs induced by the +10 D lens, -10 D lens, and FD treatments are shown graphically in Fig 3. The +10 D lens group showed the largest changes over 48 h, with the changes over the first 2 h being relatively small compared to both the changes over the later 46 h for this group and the early changes for -10 D, and FD treatment groups. Both of the latter groups also showed overshoot, larger in the case of latter group. Nonetheless all groups had largely normalized, in terms of both REs and ChT after 48 h.

### Differential BMP gene expression during recovery from +10 D lens treatment

**Fig 4:**
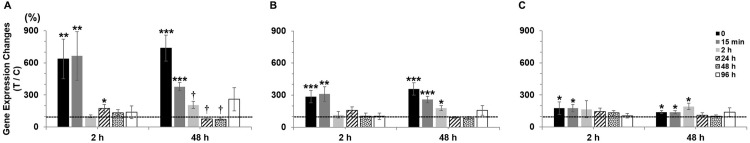
Differential gene expression for BMP2 (A), BMP4 (B), and BMP7 (C) in chick RPE over a recovery period of up to 96 h after 2 or 48 h monocular +10 D lens wear. * *p* < 0.05, ** *p* < 0.01, *** *p* < 0.001, ^†^ *p* = 0.07.

With the +10 D lens treatment, the differential gene expression patterns for BMP2 over the recovery period (0–96 h), showed both similarities and differences between the 2 and 48 h treatment groups (Fig 4 and Table 2 and S1 File).

**Table 2:** BMP gene expression in chick RPE after 2 or 48 h monocular +10 D lens treatment, and a post-treatment recovery period of up to 96 h.

	2 h Treatment Recovery			48 h Treatment Recovery		
	BMP2 (%)	BMP4 (%)	BMP7 (%)	BMP2 (%)	BMP4 (%)	BMP7 (%)
**0**	635 ± 185**	287 ± 57***	176 ± 59*	739 ± 121***	358 ± 59***	140 ± 13*
**15 min**	664 ± 226**	311 ± 68**	177 ± 34*	377 ± 40***	258 ± 31***	141 ± 14*
**2 h**	101 ± 14	116 ± 30	166 ± 79	207 ± 33^†^	179 ± 23*	192 ± 30*
**24 h**	174 ± 39*	159 ± 33	146 ± 32	75 ± 15^†^	92 ± 10	112 ± 22
**48 h**	133 ± 29	132 ± 27	132 ± 23	72 ± 14^†^	86 ± 15	102 ± 12
**96 h**	138 ± 60	103 ± 31	106 ± 22	259 ± 108	157 ± 45	140 ± 41

Data reported as mean ratios (%) of expression in treated versus control eyes ± SEMs.

* *p* < 0.05

** *p* < 0.01

*** *p* < 0.001, ^†^ *p* = 0.07.

As reported previously, 2 h of +10 D lens wear induced dramatic upregulation of BMP2 gene expression, i.e., 635 ± 185% (n = 10, *p* < 0.01, Fig 4A), and this elevation in BMP2 gene expression was sustained transiently after lens removal, to be still significant 15 min into the recovery period, i.e., 664 ± 226% (n = 8, *p* < 0.01), but lost by 2 h, with the exception of a late, apparent rebound upregulation in expression 24 h into the recovery period, i.e., 174 ± 39% (n = 17, *p* < 0.05).

With the +10 D lens, 48 h of treatment resulted in more enduring BMP2 gene expression upregulation over the recovering period than the shorter 2 h treatment (Fig 4A). Specifically, at the end of the 48 h treatment period, significant upregulation was detected, i.e., 739 ± 121% (n = 10, *p* < 0.001), and the same pattern was still in evidence, both 15 min and 2 h into the recovery period, i.e., 377 ± 40%, (n = 8, *p* < 0.001), and 207 ± 33% (n = 12, *p* = 0.07) respectively. In contrast, at two of the three later recovery timepoints, i.e., 24 and 48 h, upregulation was replaced by downregulation in treated compared to fellow eyes, i.e., 75 ± 15% (n = 9) and 72 ± 14% (n = 8) respectively (*p* = 0.07 for both cases), consistent with “overshooting”. No significant differential BMP2 gene expression was observed at the last, 96 h timepoint.

Both BMP4 and BMP7 showed similar differential gene expression patterns to that just described for BMP2, albeit smaller in magnitude (Fig 4B and 4C, and Table 2). For example, for the 2 h +10 D lens treatment, BMP4 gene expression remained elevated in treated eyes 15 min into the recovery period (311 ± 68%, *p* < 0.01), but was no longer significantly elevated 2 h into the recovery period. As with BMP2 gene expression, the longer 48 h treatment duration induced more enduring upregulation of BMP4 gene expression over the recovering period, i.e., 258 ± 31% (*p* < 0.001) and 179 ± 23% (*p* < 0.05), at 15 min and 2 h respectively. In the case of BMP7 gene expression and the 2 h treatment group, significant elevation was limited to 15 min into the recovery period (177 ± 34%, *p* < 0.05), while in the case of the 48 h treatment group, BMP7 gene expression remained elevated up to 2 h into the recovery period (192 ± 30%, *p* < 0.05).

### Differential BMP gene expression during recovery from -10 D lens treatment

**Fig 5:**
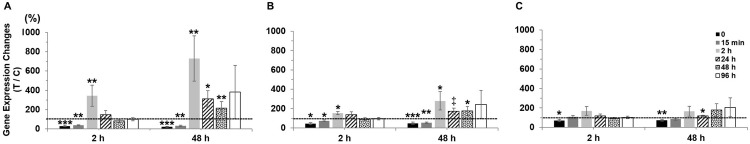
Differential gene expression of BMP2 (A), BMP4 (B), and BMP7 (C) in chick RPE over a recovery period of up to 96 h, after 2 or 48 h monocular -10 D lens treatment. * *p* < 0.05, ** *p* < 0.01, *** *p* < 0.001, ^‡^ *p* = 0.05.

With the -10 D lens worn for 2 or 48 h, BMP2 and BMP4 showed similar differential gene expression patterns, both at the end of the two treatment periods and over the respective recovery periods. Here also, BMP7 showed the smallest differential gene expression changes of the three BMPs (Fig 5 and Table 3 and S1 File).

**Table 3:** BMP gene expression in chick RPE after 2 or 48 h monocular -10 D lens treatment, and a post-treatment recovery period of up to 96 h.

	2 h Treatment Recovery			48 h Treatment Recovery		
	BMP2 (%)	BMP4 (%)	BMP7 (%)	BMP2 (%)	BMP4 (%)	BMP7 (%)
**0**	31 ± 8***	45 ± 10*	71 ± 11*	23 ± 5***	49 ± 11***	74 ± 10**
**15 min**	37 ± 5**	73 ± 8*	108 ± 14	33 ± 6**	54 ± 7**	85 ± 10
**2 h**	345 ± 109**	153 ± 17*	170 ± 45	728 ± 234**	280 ± 94*	166 ± 52
**24 h**	148 ± 42	138 ± 27	120 ± 19	312 ± 83*	172 ± 34^‡^	118 ± 7*
**48 h**	84 ± 17	90 ± 14	94 ± 8	215 ± 69**	175 ± 45*	178 ± 63
**96 h**	101 ± 16	96 ± 13	102 ± 13	383 ± 276	243 ± 144	206 ± 98

Data reported as mean ratios (%) of expression in treated versus control eyes ± SEMs.

* *p* < 0.05

** *p* < 0.01

*** *p* < 0.001

^‡^ *p* = 0.05.

When the gene expression profiles for the -10 D lens groups are compared to those of the +10 D lens groups, a number of differences are apparent, including the direction of initial gene expression changes for the two -10 D lens groups (2 & 48 h), which is opposite to that described for the +10 D lens groups. For BMP2 gene expression, downregulation in treated compared to contralateral fellow eyes was observed with the -10 D lens treatment, after both 2 and 48 h of lens wear (31 ± 8%, n = 10, *p* < 0.001; 23 ± 5%, n = 10, *p* < 0.001 respectively. Fig 5A). This pattern of downregulation also persisted for up to 15 min after removal of the -10 D lens, for both the 2 h treatment group i.e. 37 ± 5% (n = 10, *p* < 0.01) and the 48 h treatment group, i.e., 33 ± 6% (n = 9, *p* < 0.01). When allowed longer recovery, the direction of gene expression changes reversed, with downregulation being replaced by upregulation by 2 h into the recovery period, and persisting over a variable period, being more enduring with the longer initial treatment duration (Fig 5 and Table 3). Specifically, by 2 h into the recovery period, gene expression was upregulated to 345 ± 109% (n = 8, *p* < 0.01) for the 2 h group, and to 728 ± 234% (n = 12, *p* < 0.01) for the 48 h group. However, gene expression upregulation was short-lived for the 2 h treatment group, with treated and fellow eyes recording similar gene expression levels 24 h into the recovery period (148 ± 42%, n = 10, *p* > 0.05). In contrast, for the 48 h treatment group, significant gene expression upregulation was still apparent 24 and 48 h into the recovery period, i.e., 312 ± 83% (n = 10, *p* < 0.05) and 215 ± 69% (n = 13, *p* < 0.01), respectively, although gene expression had normalized by 96 h into the recovery period, when treated and fellow eyes recorded similar gene expression levels.

The differential gene expression patterns for BMP4 were very similar to those just described for BMP2, for both 2 and 48 h lens treatment groups (Fig 5B and Table 3). Thus BMP4 gene expression downregulation was observed after both 2 and 48 h of -10 D lens treatment, i.e., 45 ± 10% (*p* < 0.05), and 49 ± 11% (*p* < 0.001), with downregulation persisting up to 15 min after lens removal for both 2 and 48 h groups, i.e., 73 ± 8% (*p* < 0.05) and 54 ± 7% (*p* < 0.01) respectively. Later into the recovery period, as observed with BMP2 gene expression, downregulation in treated eyes was replaced by upregulation. Specifically, by 2 h into the recovery period, gene expression had increased to 153 ± 17% (*p* < 0.05) and 280 ± 94% (*p* < 0.05), for the 2 and 48 h treatment groups, respectively. For BMP4 and the 2 h treatment group, differential gene expression was not significantly altered beyond this recovery timepoint, e.g., 138 ± 27% (*p* > 0.05), for the 24 h time point, while in contrast, for the 48 h treatment group, BMP4 gene expression upregulation persisted out to 48 h into the recovery period, i.e., 172 ± 34% (*p* = 0.05) at 24 h, and 175 ± 45% (*p* < 0.05) at 48 h, but was no longer detectible at the 96 h timepoint.

In the case of BMP7, both 2 and 48 h treatment groups showed significant gene expression downregulation, albeit smaller in magnitude than recorded for BMP2 and BMP4 (2 h: 71 ± 11%, *p* < 0.05; 48 h: 74 ± 10%, *p* < 0.01). Accordingly, gene expression upregulation over the recovery period reached statistical significance only for the 48 h treatment group and 24 h of recovery (118 ± 7%, *p* < 0.05).

### Differential BMP gene expression during recovery from form-deprivation treatment

**Fig 6:**
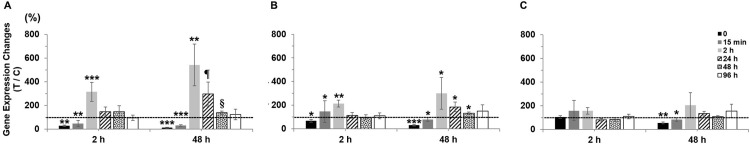
Differential gene expression of BMP2 (A), BMP4 (B), and BMP7 (C) in chick RPE over a recovery period of up to 96 h after 2 or 48 h monocular form-deprivation treatment. * *p* < 0.05, ** *p* < 0.01, *** *p* < 0.001, ^¶^ *p* = 0.06, ^§^ *p* = 0.09.

The differential gene expression patterns for BMP2 and BMP4 recorded with the diffuser (form-deprivation, FD) treatment are similar to those just described for the -10 D lens treatment, for both 2 and 48 h treatment durations and across the recovery period (Fig 6 and Table 4 and S1 File). On the other hand, subtle treatment-related differences in the BMP7 gene expression profiles are apparent, with significant differential BMP7 gene expression changes limited to the 48 h FD treatment group and just one recovery timepoint.

**Table 4:** BMP gene expression in chick RPE after 2 or 48 h monocular form-deprivation treatment and a post-treatment recovery period of up to 96 h.

	2 h Treatment Recovery			48 h Treatment Recovery		
	BMP2 (%)	BMP4 (%)	BMP7 (%)	BMP2 (%)	BMP4 (%)	BMP7 (%)
**0**	27 ± 6**	68 ± 13*	103 ± 12	12 ± 3***	31 ± 6***	56 ± 9**
**15 min**	47 ± 25**	147 ± 92*	158 ± 85	30 ± 7***	80 ± 16*	83 ± 14*
**2 h**	315 ± 80***	214 ± 28**	156 ± 28	542 ± 176**	301 ± 134*	205 ± 105
**24 h**	148 ± 38	113 ± 24	81 ± 11	296 ± 102^¶^	186 ± 40*	133 ± 20
**48 h**	146 ± 52	98 ± 22	85 ± 16	139 ± 15^§^	131 ± 12*	107 ± 9
**96 h**	95 ± 21	111 ± 25	110 ± 20	124 ± 46	149 ± 53	156 ± 56

Data reported as mean ratios (%) of expression in treated versus control eyes ± SEMs.

* *p* < 0.05

** *p* < 0.01

*** *p* < 0.001

^¶^ *p* = 0.06

^§^ *p* = 0.09.

For BMP2, both 2 and 48 h of FD treatment resulted in gene expression downregulation in treated compared to fellow eyes, i.e., to 27 ± 6% (n = 10, *p* < 0.01) and 12 ± 3% (n = 10, *p* < 0.001) respectively (Fig 6A). Here also, as with the -10 D lens treatment, this downregulation pattern was still evident 15 min after the removal of diffusers, for both 2 and 48 h treatment groups, i.e., 47 ± 25% (n = 9, *p* < 0.01) and 30 ± 7%, (n = 11, *p* < 0.001) respectively. Thereafter, the direction of BMP2 gene expression changed, with significant upregulation recorded after 2 h of recovery, for both 2 and 48 h treatment groups, i.e., 315 ± 80% (n = 10, *p* < 0.001) and 542 ± 176% (n = 9, *p* < 0.01), respectively. Beyond 2 h of recovery, persistent, significant upregulation was only recorded for the 48 h treatment group, i.e., 296 ± 102% (n = 12, *p* = 0.06) and 139 ± 15% (n = 12, *p* = 0.09), after 24 and 48 h of recovery respectively, with gene expression normalizing in treated eyes by 96 h into the recovery period, i.e., 124 ± 46% (n = 10, *p* > 0.05). For the shorter, 2 h treatment group, this pattern of upregulation had disappeared by 24 h into the recovery period, i.e., 148 ± 38% (n = 12, *p* > 0.05).

In the case of BMP4, as with BMP2, both 2 h and 48 of FD treatment induced downregulation, i.e. 68 ± 13% (*p* < 0.05) and 31 ± 6% (*p* < 0.001) respectively (Fig 6B). Likewise, the direction of BMP4 gene expression rapidly reversed after removal of the diffusers. After the 2 h treatment, upregulation was recorded 15 min and 2 h into the recovery period, to 147 ± 92% (*p* < 0.05) and 214 ± 28% (*p* < 0.01) respectively, while no significant differential gene expression was detected by 24 h into the recovery period (113 ± 24%, *p* > 0.05). After the longer, 48 h FD treatment, downregulation persisted for 15 min into the recovery period, i.e., 80 ± 16% (*p* < 0.05), being replaced by 2 h with significant upregulation, which persisted out to 48 h into the recovery period, i.e. 301 ± 134% (*p* < 0.05), 186 ± 40% (*p* < 0.05), and 131 ± 12% (*p* < 0.05) at 2, 24, and 48 h, respectively. No significant differential gene expression of BMP4 was observed at the last, 96 h recovery timepoint.

In the case of BMP7, significant differential gene expression changes were limited to the longer 48 h FD treatment, and here only at the end of the treatment period and 15 min into the recovery period, i.e., 56 ± 9% (*p* < 0.01) and 83 ± 14% (*p* < 0.05) respectively (Fig 6C).

### Correlation between BMP2 gene expression and choroid thickness changes during recovery

**Fig 7:**
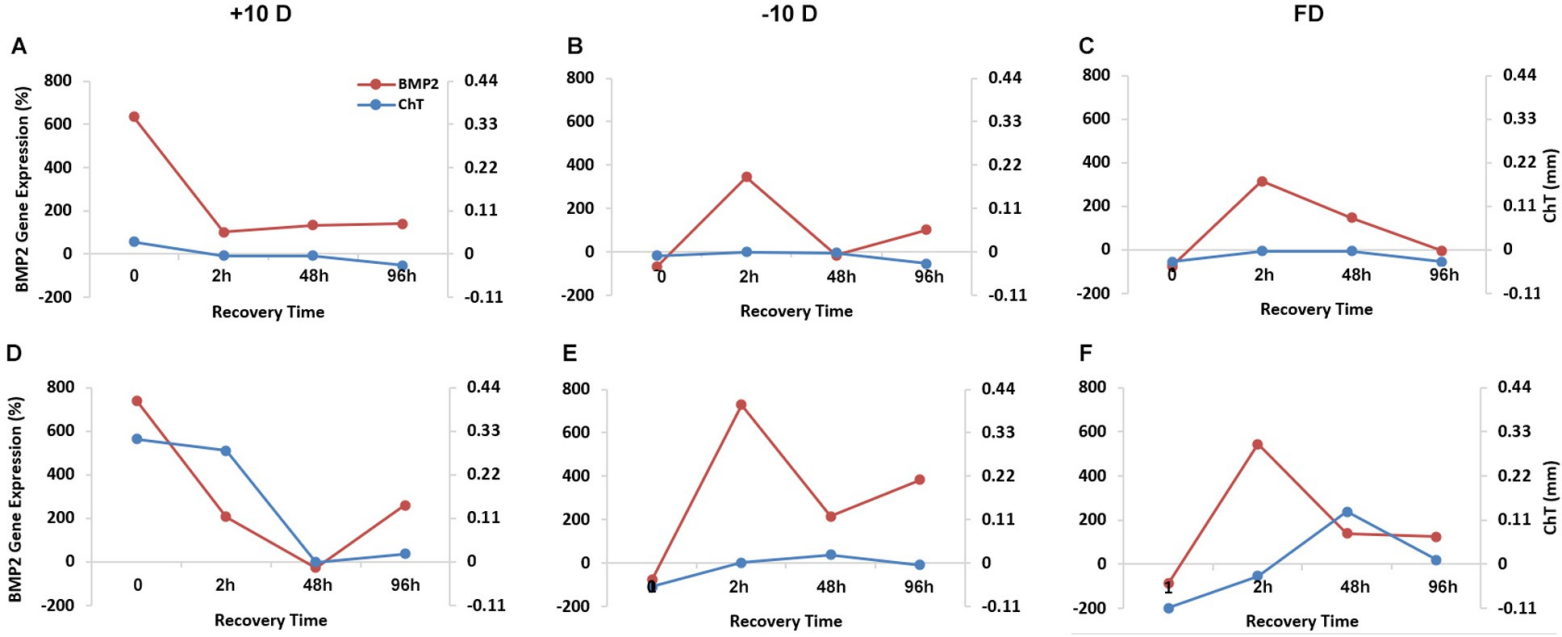
Relationship between BMP2 gene expression levels and ChT during recovery from +10 D (A, D), -10 D (B, E), and FD (C, F) treatments, applied for 2 h (A-C) or 48 h (D-F). Relative BMP2 gene expression levels (treated/control eyes) are plotted (Y-axis on left), along with interocular differences in ChT (Y-axis on right).

For the 2 h treatment groups, choroid thickness changes did not achieve statistical significance for any of the three visual manupulations, +10 D, -10 D and FD, despite changes in BMP2 gene expression, which in all three cases, showed rapid normalizaton after their termination, and in the case of the -10 D and FD treatments, overshoot (Fig 7A–7C). Of the three 48 h treatment groups, the +10 D lens group stood apart, with both BMP2 gene expression levels and choroid thickness tending to normalize over a similar time frame (Fig 7D). In contrast, for both -10 D and FD treatments, BMP2 gene expression levels showed more dynamic changes over the recovery period, increasing and then decreasing (Fig 7E and 7F), although only the FD group showed significant overshoot in recovery-related choroid thickness changes, which also lagged behind temporally, the gene expression changes (Fig 7F).

## Discussion

In our previous studies of chick RPE, we demonstrated robust, bidirectional regulation of three BMP genes, BMP2, BMP4, and BMP7, with the direction of regulation changing in accord with the sign of defocus [28, 29]. Specifically, BMP2 gene expression was upregulated with imposed myopic defocus (imposed by positive lenses) and downregulated with hyperopic defocus (imposed by negative lenses). With form-deprivation, which, along with imposed hyperopic defocus, accelerates eye growth, BMP2 gene expression was also downregulated [30, 31]. In the case of all three visual manipulations, the induced changes in BMP gene expression occurred rapidly. For example, with imposed myopic defocus, the induced upregulation of BMP2 gene expression was detectable after as little as 15 minutes, before detectable changes in axial length [30]. The minimum treatment duration inducing significant downregulation of BMP2 gene expression with imposed hyperopic defocus and form-deprivation was 2 h, longer than for imposed myopic defocus, albeit still relatively short [28–30].

The findings of the studies reported here confirm those of our previous studies, as summarized above, specifically, that the expressions of both BMP2 and BMP4 genes are differential and tightly regulated by the defocus status of the eye. This is despite the visual manipulations, such as optical defocus, being limited to normal eyes in our earlier studies, while the current study included eyes recovering from induced refractive errors and thus changes in one or more of their ocular components, including the thickness of the choroid, which is immediately adjacent to the RPE. Such differences might be expected to lead to altered or more complex patterns of RPE BMP gene expression changes. Interpretation of gene expression changes over the recovery periods, i.e., after the termination of the inducing treatments, is further complicated by the relatively small and more labile nature of changes in eyes with induced myopia compared to hyperopia. By way of example, with the +10 D lens worn for 48 h, hyperopia was still detectible up to 2 h after its removal, i.e., into the recovery period. For the same 48 h treatment, BMP2 gene expression was initially increased in the RPE of treated eyes, but then rapidly declined within just 2 h of recovery, to be minimally elevated after 24 h. The early changes in BMP2 mRNA levels in treated eyes, i.e., at 15 min and 2 h into the recovery period, presumably reflect a combination of hyperopic defocus-induced downregulation of BMP2 gene expression and degradation of recently synthesized mRNA. BMP2 mRNA levels continued to decline in treated eyes to approximately match the levels in contralateral control eyes by 48 h into the recovery period. Choroidal thickening, a product of the initially imposed defocus, was still detectible 2 h into the recovery period, although continued to decline over time, such that the choroids of treated eyes approximately matched in thickness, those of their fellows, 48 h into the recovery period, in parallel with the normalization of gene expression. In the case of the -10 D lens, both 2 h and 48 h of treatment resulted in detectible BMP2 gene expression downregulation in treated eyes, followed by upregulation in the recovery period, persisting for up to 48 h. The latter pattern is consistent with the robust nature of the response to myopic defocus, originating in this case from the ocular dimensional changes induced by wearing a -10 D lens. With the longer 48 h induction period, previously treated eyes would have experienced myopic defocus for at least 2 h into the recovery period, and under these conditions, ChT returned to normal within 2 h before slightly overshooting, i.e., increasing. That the effect of the initial inducing lens treatment was to downregulate BMP2 gene expression also simplifies the picture at a molecular level, there being no surplus BMP2 mRNA to degrade in this case. Thus the data directly reflect altered gene expression. Similar patterns of BMP2 gene expression changes over time were observed with the FD treatment, with similar explanations likely to hold.

Changes in both refractive errors and ocular dimensions consistent with recovery after the termination of visual manipulations known to affect the former have been well documented across a range of animal models including chicks, tree shrews, guinea pigs, marmosets and monkeys [8, 16, 18, 32–43]. Thus in chicks, sufficiently long exposure to positive lenses induces hyperopia, a byproduct of choroid thickening and slowed ocular elongation. In the absence of the inducing lenses, the retinal experience of hyperopia triggers the opposite responses, choroidal thinning and acceleration of ocular elongation, and ultimately normalizing ChT and axial length dimensions, with the net myopic shift in refractive error ultimately correcting for the induced refractive error [16]. Similarly, when myopia-inducing treatments, either negative lenses or form depriving diffusers, are removed, the myopic defocus experienced by the retina leads to choroidal thickening and slowed axial elongation, linked to hyperopic shifts in refraction [16, 32, 33]. Our working hypothesis for the experiments described here was that the altered ocular growth patterns underlying the recovery of normal ocular dimensions are mediated by the same retina-sclera signalling cascades involved in responses of previously untreated eyes to lens-imposed defocus, with changes in BMP gene expression in the RPE serving as a biomarker of the direction of growth changes [8, 23, 44–47]. Our findings are consistent with this hypothesis, although examples of over-shoot in gene expression changes during recovery from the changes induced by the +10 D lenses also hint at differences. In addition to the need in the latter case to degrade overexpressed mRNA, the observed overshoot during recovery may point to additional non-visual, developmental influences on the growth of the eyes, as known for other organs of young animals, such as circulating growth hormones [8]. Among previous investigations of myopia-inducing and recovery responses involving animal models, various patterns of changes in gene and/or protein expression in the retina, RPE, choroid, and/or sclera have been described [48–55]. Some of these studies suggested that myopia-inducing and inhibiting signalling pathways may share little in common [20, 56].

The results of this study have potential clinical implications, assuming similar RPE gene expression changes mediate defocus-mediated changes in choroidal thickness and/or ocular growth changes in humans. Our recently reported finding of decreases in RPE-BMP2 gene expression in young guinea pigs in response to imposed hyperopic defocus offers supporting evidence for the generalizability of this finding [57]. Thus at a clinical level, that the gene expression changes induced by imposed myopic defocus (i.e., with positive lenses) were more enduring than those resulting from myopia-genic conditions (e.g., with negative lenses), supports as a strategy to slow myopia progression, regular interruption to near work activities, at least for short periods, assuming lags of accommodation are a contributing driver [58]. The success of clinical myopia control strategies that seek to impose myopic defocus on at least part of the retina, for part of the day, e.g., using multifocal soft contact and orthokeratology lenses, is also consistent with the findings reported herein [7].

In summary, our current study provides further evidence for dynamic, defocus-driven, bidirectional regulation in chick RPE of BMP gene expression, with changes in BMP2 and BMP4 genes within this BMP family being the most robust. Together with findings from our previous studies in chick involving induced myopia and hyperopia, the directional consistency of changes in gene expression within this BMP family, choroidal thickness and rates of ocular elongation, with upregulation tied to choroidal thickening and slowed elongation, and vice versa, further suggests roles for these growth factors, beyond serving as biomarkers of ocular growth trends. Together, these studies provide strong supporting evidence for a role of RPE-derived BMPs in eye growth regulation, which warrant follow-up investigations of their potential application as myopia control therapies.

## Supporting information

S1 File Refractive errors, choroidal thickness, and gene expression changes in chick RPE over a recovery period of up to 96 h after 2 or 48 h monocular +10 & -10 D lens wear or FD treatment. (DOCX)
