# Supplementary material for: Dynamic BMP gene expression regulation in chick RPE during recovery from short term optical defocus and form-deprivation
Source: PLoS One. 2024 Oct 11;19(10):e0311505. doi: 10.1371/journal.pone.0311505 (PMC11469538; doi:10.1371/journal.pone.0311505)
Supplement: S1 File — (DOCX) [file pone.0311505.s001.docx]

**Title:** Dynamic BMP Gene Expression Regulation in Chick RPE during Recovery from Short Term Optical Defocus and Form-Deprivation

**PONE-D-23-26130**

1. **Refractive Errors (D) after recovery periods of up to 96 h after 2 or 48 h monocular optical defocus or form deprivation (FD) treatment**

+10D, 2 h

| Sample# | Treated eye | | | | | Control eye | | | | |
| --- | --- | --- | --- | --- | --- | --- | --- | --- | --- | --- |
|  | Baseline | Wear 2h | Rec 2h | Rec 48h | Rec 96h | Baseline | Wear 2h | Rec 2h | Rec 48h | Rec 96h |
| 1 | 5.75 | 7.25 | 6.5 | 5.25 | 5.25 | 5.5 | 5.5 | 5.5 | 5.25 | 5.25 |
| 2 | 4.75 | 8.25 | 7 | 4.75 | 4.75 | 4.75 | 5 | 5 | 5 | 5 |
| 3 | 4.5 | 8.5 | 7.25 | 5.25 | 5 | 4.5 | 5 | 4.75 | 4.75 | 4.75 |
| 4 | 4.5 | 7.25 | 5.75 | 4.75 | 4.75 | 5.25 | 5 | 5 | 5 | 5 |
| 5 | 5.5 | 7.75 | 6.25 | 5.5 | 5.5 | 5.25 | 5 | 5 | 5.5 | 5.5 |
| 6 | 5.25 | 6.75 | 6.25 | 5.5 | 5.25 | 5.25 | 5.25 | 5.25 | 5.25 | 5.25 |
| 7 | 5.75 | 7 | 6 | 5.75 | 5.75 | 5.5 | 5.5 | 5.25 | 5.5 | 5.5 |
| 8 | 5.5 | 7.5 | 6.5 | 5.5 | 5.25 | 5.25 | 5.5 | 5.25 | 5.25 | 5.25 |

-10D, 2 h

| Sample# | Treated eye | | | | | Control eye | | | | |
| --- | --- | --- | --- | --- | --- | --- | --- | --- | --- | --- |
|  | Baseline | Wear 2h | Rec 2h | Rec 48h | Rec 96h | Baseline | Wear 2h | Rec 2h | Rec 48h | Rec 96h |
| 1 | 5.5 | 4.5 | 5 | 5 | 4.75 | 5.5 | 5.5 | 5.5 | 5.25 | 5.25 |
| 2 | 5.25 | 3.75 | 4.25 | 5.25 | 5.5 | 5 | 4.75 | 4.75 | 5 | 5 |
| 3 | 5.25 | 4 | 5 | 5 | 5 | 5 | 5.25 | 5 | 5 | 5 |
| 4 | 5.75 | 4.75 | 4.75 | 5 | 5.5 | 5.5 | 5.75 | 5.75 | 5.5 | 5.5 |
| 5 | 5 | 4 | 5 | 5 | 5 | 5 | 5.25 | 5 | 5 | 5 |
| 6 | 4.75 | 4 | 4.75 | 5 | 5 | 4.75 | 4.75 | 5 | 5 | 5 |
| 7 | 5 | 4 | 5 | 5.25 | 5.25 | 5.5 | 5.5 | 5.25 | 5.5 | 5.25 |
| 8 | 5 | 4 | 5 | 5 | 5 | 4.75 | 4.75 | 4.75 | 4.75 | 4.75 |

FD, 2 h

| Sample# | Treated eye | | | | | Control eye | | | | |
| --- | --- | --- | --- | --- | --- | --- | --- | --- | --- | --- |
|  | Baseline | Wear 2h | Rec 2h | Rec 48h | Rec 96h | Baseline | Wear 2h | Rec 2h | Rec 48h | Rec 96h |
| 1 | 5 | 4 | 5 | 5.25 | 5.25 | 5 | 5 | 5 | 5 | 5 |
| 2 | 4.75 | 3.75 | 5 | 5 | 5 | 5 | 4.75 | 5 | 5 | 5 |
| 3 | 5.5 | 4.25 | 5 | 5.5 | 5.25 | 4.75 | 5 | 5 | 4.75 | 4.75 |
| 4 | 5.5 | 4.75 | 5 | 5.25 | 5 | 5 | 5 | 5 | 5 | 5 |
| 5 | 5.5 | 4 | 5.25 | 5.5 | 5.25 | 5 | 5.25 | 5 | 5 | 5 |
| 6 | 4.75 | 4 | 4.75 | 5 | 5 | 4.5 | 4.75 | 4.75 | 4.75 | 4.75 |
| 7 | 4.75 | 3.75 | 4.75 | 5 | 4.75 | 4.75 | 4.75 | 4.75 | 4.75 | 4.75 |

1. **Choroid Thickness (mm) after recovery periods of up to 96 h after 2 or 48 h monocular optical defocus or form deprivation (FD) treatment**

+10D, 2 h

| Sample# | Treated eye | | | | | Control eye | | | | |
| --- | --- | --- | --- | --- | --- | --- | --- | --- | --- | --- |
|  | Baseline | Wear 2h | Rec 2h | Rec 48h | Rec 96h | Baseline | Wear 2h | Rec 2h | Rec 48h | Rec 96h |
| 1 | 0.19079 | 0.22051 | 0.18887 | 0.22051 | 0.20421 | 0.17833 | 0.17545 | 0.15819 | 0.15255 | 0.22147 |
| 2 | 0.19175 | 0.25695 | 0.19367 | 0.13976 | 0.20134 | 0.14957 | 0.18504 | 0.19271 | 0.13806 | 0.18664 |
| 3 | 0.20805 | 0.35378 | 0.27229 | 0.15915 | 0.13518 | 0.20421 | 0.17737 | 0.16203 | 0.15244 | 0.16704 |
| 4 | 0.19654 | 0.27782 | 0.21476 | 0.15148 | 0.19271 | 0.17471 | 0.22051 | 0.20133 | 0.18504 | 0.24544 |
| 5 | 0.17449 | 0.20121 | 0.18792 | 0.14477 | 0.11409 | 0.14285 | 0.17300 | 0.18408 | 0.17066 | 0.13710 |
| 6 | 0.14094 | 0.21380 | 0.15724 | 0.14861 | 0.20112 | 0.13518 | 0.16874 | 0.16682 | 0.17641 | 0.16491 |
| 7 | 0.22148 | 0.36912 | 0.37327 | 0.29817 | 0.14285 | 0.25503 | 0.22435 | 0.37104 | 0.20517 | 0.19175 |
| 8 | 0.31159 | 0.32129 | 0.28667 | 0.25215 |  | 0.19516 | 0.25215 | 0.27996 | 0.17353 |  |

-10D, 2 h

| Sample# | Treated eye | | | | | Control eye | | | | |
| --- | --- | --- | --- | --- | --- | --- | --- | --- | --- | --- |
|  | Baseline | Wear 2h | Rec 2h | Rec 48h | Rec 96h | Baseline | Wear 2h | Rec 2h | Rec 48h | Rec 96h |
| 1 | 0.28571 | 0.33077 | 0.31926 | 0.16682 | 0.20421 | 0.32981 | 0.32406 | 0.32789 | 0.20709 | 0.20613 |
| 2 | 0.14232 | 0.12464 | 0.12656 | 0.13721 | 0.20038 | 0.12751 | 0.16778 | 0.14190 | 0.15819 | 0.21955 |
| 3 | 0.20997 | 0.17545 | 0.20805 | 0.19463 | 0.16491 | 0.33918 | 0.26419 | 0.23298 | 0.15340 | 0.22435 |
| 4 | 0.16778 | 0.15340 | 0.20112 | 0.20230 | 0.17833 | 0.18408 | 0.19079 | 0.18408 | 0.18025 | 0.23681 |
| 5 | 0.30776 | 0.24544 | 0.23351 | 0.18408 | 0.15436 | 0.18025 | 0.16011 | 0.26078 | -.19846 | 0.21093 |
| 6 | 0.30584 | 0.25023 | 0.27996 | 0.20709 | 0.12943 | 0.32751 | 0.32214 | 0.29050 | 0.24065 | 0.17258 |
| 7 | 0.16874 | 0.19367 | 0.14381 | 0.14381 | 0.14477 | 0.14477 | 0.14573 | 0.15819 | 0.13135 | 0.16682 |
| 8 | 0.92136 | 0.49835 | 0.79877 | 0.60716 | 0.00511 | 0.21093 | 0.21093 | 0.11697 | 0.20421 | 0.18504 |

FD, 2 h

| Sample# | Treated eye | | | | | Control eye | | | | |
| --- | --- | --- | --- | --- | --- | --- | --- | --- | --- | --- |
|  | Baseline | Wear 2h | Rec 2h | Rec 48h | Rec 96h | Baseline | Wear 2h | Rec 2h | Rec 48h | Rec 96h |
| 1 | 0.17929 | 0.17162 | 0.18238 | 0.20517 | 0.18696 | 0.13998 | 0.16984 | 0.16778 | 0.17641 | 0.19613 |
| 2 | 0.19079 | 0.18493 | 0.20038 | 0.21476 | 0.11505 | 0.19175 | 0.13806 | 0.17258 | 0.18686 | 0.17641 |
| 3 | 0.25982 | 0.16203 | 0.30488 | 0.18312 | 0.13998 | 0.20421 | 0.20326 | 0.21188 | 0.14765 | 0.23489 |
| 4 | 0.21135 | 0.17833 | 0.16107 | 0.19846 | 0.186 | 0.19345 | 0.20326 | 0.17385 | 0.19079 | 0.21093 |
| 5 | 0.24352 | 0.23394 | 0.20613 | 0.21764 | 0.19846 | 0.19079 | 0.21572 | 0.17641 | 0.20901 | 0.20230 |
| 6 | 0.32598 | 0.31447 | 0.32693 | 0.31447 | 0.30850 | 0.25993 | 0.33748 | 0.32693 | 0.29817 | 0.20805 |
| 7 | 0.23202 | 0.25982 | 0.24928 | 0.28123 | 0.27037 | 0.22828 | 0.23777 | 0.19367 | 0.19079 | 0.1812 |

1. **BMP gene expression in chick RPE over a recovery period of up to 96 h after 2 or 48 h monocular +10 D lens wear (gene expression level compared against GAPDH)**

+10D, 2h treatment; BMP2

|  | Rec 15min | | Rec 2h | | Rec 24h | | Rec 48h | | Rec 96h | |
| --- | --- | --- | --- | --- | --- | --- | --- | --- | --- | --- |
| Sample# | Treated | Control | Treated | Control | Treated | Control | Treated | Control | Treated | Control |
| 1 | 2.40502 | 0.43166 | 0.44669 | 0.53936 | 0.85569 | 0.41758 | 0.56854 | 0.72305 | 0.55332 | 0.46601 |
| 2 | 2.89557 | 0.13539 | 0.70674 | 1.16674 | 0.67515 | 0.47040 | 0.54428 | 0.45177 | 0.34706 | 0.08121 |
| 3 | 3.31030 | 0.59819 | 0.91222 | 1.30023 | 0.55420 | 0.19409 | 2.26593 | 1.56957 | 0.15508 | 0.30436 |
| 4 | 2.25121 | 0.53823 | 1.03401 | 0.87719 | 0.13546 | 0.10460 | 0.36788 | 0.63941 | 0.13886 | 0.48040 |
| 5 | 3.19629 | 1.10884 | 0.34076 | 0.22500 | 0.67061 | 0.35980 | 0.44196 | 0.25164 | 0.18133 | 0.26583 |
| 6 | 1.71895 | 0.83561 | 0.30545 | 0.63241 | 0.54711 | 0.41450 | 0.48309 | 0.25824 | 1.10557 | 0.81350 |
| 7 | 1.08510 | 0.45368 | 0.59989 | 0.52259 | 1.65450 | 1.61648 | 0.24481 | 0.20619 | 0.26556 | 0.36617 |
| 8 | 0.46802 | 0.05126 | 0.78268 | 0.44662 | 0.41347 | 0.55648 | 0.21829 | 0.56925 | 0.21554 | 0.23558 |
| 9 |  |  | 0.86256 | 1.00774 | 0.45235 | 0.62476 | 0.52524 | 0.85630 | 0.39977 | 0.31963 |
| 10 |  |  |  |  | 0.47897 | 0.54491 | 0.24458 | 0.07068 | 0.08278 | 0.42473 |
| 11 |  |  |  |  | 1.16484 | 0.50809 |  |  |  |  |
| 12 |  |  |  |  | 0.18683 | 0.18839 |  |  |  |  |
| 13 |  |  |  |  | 1.03174 | 0.48500 |  |  |  |  |
| 14 |  |  |  |  | 0.28803 | 0.22047 |  |  |  |  |
| 15 |  |  |  |  | 0.87374 | 0.11874 |  |  |  |  |
| 16 |  |  |  |  | 0.55991 | 0.41666 |  |  |  |  |
| 17 |  |  |  |  | 0.00756 | 0.37251 |  |  |  |  |

+10D, 48h treatment; BMP2

|  | Rec 15min | | Rec 2h | | Rec 24h | | Rec 48h | | Rec 96h | |
| --- | --- | --- | --- | --- | --- | --- | --- | --- | --- | --- |
| Sample# | Treated | Control | Treated | Control | Treated | Control | Treated | Control | Treated | Control |
| 1 | 2.30061 | 0.56860 | 2.55954 | 1.02892 | 0.20085 | 1.30164 | 0.55209 | 1.04790 | 0.35795 | 0.03509 |
| 2 | 0.67803 | 0.28750 | 0.04592 | 0.25539 | 0.68283 | 0.97122 | 0.27288 | 0.27114 | 0.15614 | 0.16619 |
| 3 | 1.40597 | 0.30196 | 0.22119 | 0.10541 | 0.42206 | 1.14012 | 0.30651 | 0.98421 | 0.40808 | 0.07864 |
| 4 | 2.11671 | 0.70037 | 0.32557 | 0.25911 | 0.19527 | 0.33864 | 0.32557 | 0.25911 | 0.27895 | 0.54712 |
| 5 | 2.38125 | 0.66810 | 2.38125 | 0.66810 | 0.38974 | 0.48812 | 0.16932 | 0.42340 | 0.47693 | 0.43499 |
| 6 | 1.71034 | 0.70941 | 0.43757 | 0.34172 | 0.12674 | 0.18855 | 0.33042 | 0.35907 | 0.72037 | 0.23694 |
| 7 | 1.42011 | 0.29713 | 0.18488 | 0.13197 | 0.29064 | 0.16262 | 0.23699 | 0.21857 | 0.37404 | 0.72689 |
| 8 | 0.85996 | 0.16104 | 0.18106 | 0.07093 | 0.36121 | 0.42850 | 0.07628 | 0.30621 | 0.65433 | 0.87880 |
| 9 |  |  | 0.36812 | 0.10092 | 0.39935 | 0.47585 |  |  | 0.49172 | 0.44472 |
| 10 |  |  | 0.61612 | 0.16677 |  |  |  |  |  |  |
| 11 |  |  | 0.13281 | 0.08467 |  |  |  |  |  |  |
| 12 |  |  | 0.17188 | 0.16139 |  |  |  |  |  |  |

+10D, 2h treatment; BMP4

|  | Rec 15min | | Rec 2h | | Rec 24h | | Rec 48h | | Rec 96h | |
| --- | --- | --- | --- | --- | --- | --- | --- | --- | --- | --- |
| Sample# | Treated | Control | Treated | Control | Treated | Control | Treated | Control | Treated | Control |
| 1 | 0.20954 | 0.07778 | 0.11838 | 0.12628 | 0.19376 | 0.11295 | 0.13800 | 0.12500 | 0.13810 | 0.18501 |
| 2 | 0.45765 | 0.06713 | 0.13552 | 0.13344 | 0.16081 | 0.11481 | 0.11812 | 0.11821 | 0.12046 | 0.03230 |
| 3 | 0.35212 | 0.12412 | 0.12812 | 0.16875 | 0.11346 | 0.04187 | 0.33841 | 0.26438 | 0.07324 | 0.10397 |
| 4 | 0.30744 | 0.06433 | 0.15090 | 0.16001 | 0.05045 | 0.06387 | 0.09659 | 0.13979 | 0.09656 | 0.19288 |
| 5 | 0.43330 | 0.26949 | 0.05902 | 0.01667 | 0.12466 | 0.11115 | 0.19391 | 0.14881 | 0.08700 | 0.13434 |
| 6 | 0.25554 | 0.25669 | 0.14637 | 0.27170 | 0.15997 | 0.11644 | 0.14063 | 0.09593 | 0.29148 | 0.27825 |
| 7 | 0.13586 | 0.07974 | 0.21073 | 0.22578 | 0.11050 | 0.11320 | 0.13447 | 0.09005 | 0.09053 | 0.10526 |
| 8 | 0.07514 | 0.02174 | 0.03810 | 0.04171 | 0.11647 | 0.16547 | 0.07606 | 0.20282 | 0.12174 | 0.14376 |
| 9 |  |  | 0.10732 | 0.12507 | 0.12297 | 0.14101 | 0.11877 | 0.13214 | 0.16586 | 0.17536 |
| 10 |  |  |  |  | 0.08892 | 0.10008 | 0.06991 | 0.01954 | 0.04033 | 0.13798 |
| 11 |  |  |  |  | 0.23291 | 0.10639 |  |  |  |  |
| 12 |  |  |  |  | 0.16438 | 0.08841 |  |  |  |  |
| 13 |  |  |  |  | 0.06453 | 0.05976 |  |  |  |  |
| 14 |  |  |  |  | 0.14385 | 0.02166 |  |  |  |  |
| 15 |  |  |  |  | 0.19836 | 0.15320 |  |  |  |  |
| 16 |  |  |  |  | 0.00336 | 0.13202 |  |  |  |  |
| 17 |  |  |  |  | 0.08704 | 0.06327 |  |  |  |  |

+10D, 48h treatment; BMP4

|  | Rec 15min | | Rec 2h | | Rec 24h | | Rec 48h | | Rec 96h | |
| --- | --- | --- | --- | --- | --- | --- | --- | --- | --- | --- |
| Sample# | Treated | Control | Treated | Control | Treated | Control | Treated | Control | Treated | Control |
| 1 | 0.35605 | 0.14463 | 0.27914 | 0.17915 | 0.06444 | 0.09372 | 0.08989 | 0.10726 | 0.13589 | 0.02787 |
| 2 | 0.10573 | 0.05195 | 0.02245 | 0.08740 | 0.10505 | 0.09930 | 0.06913 | 0.04433 | 0.04341 | 0.03682 |
| 3 | 0.20303 | 0.11459 | 0.11030 | 0.03834 | 0.06123 | 0.08641 | 0.08528 | 0.16616 | 0.11995 | 0.05054 |
| 4 | 0.34628 | 0.11788 | 0.08383 | 0.08026 | 0.08641 | 0.10181 | 0.06654 | 0.04918 | 0.17718 | 0.18241 |
| 5 | 0.28237 | 0.07278 | 0.07561 | 0.02919 | 0.05124 | 0.12017 | 0.04760 | 0.09787 | 0.16871 | 0.19279 |
| 6 | 0.40331 | 0.29631 | 0.06621 | 0.05384 | 0.06538 | 0.04543 | 0.08371 | 0.09327 | 0.12930 | 0.08188 |
| 7 | 0.20388 | 0.05636 | 0.04969 | 0.03234 | 0.11076 | 0.09880 | 0.06095 | 0.06880 | 0.08206 | 0.13450 |
| 8 | 0.18684 | 0.07190 | 0.03680 | 0.01675 | 0.07434 | 0.08146 | 0.04948 | 0.13590 | 0.12598 | 0.14034 |
| 9 |  |  | 0.09557 | 0.03572 | 0.09984 | 0.09015 |  |  | 0.09822 | 0.12531 |
| 10 |  |  | 0.20214 | 0.08534 |  |  |  |  |  |  |
| 11 |  |  | 0.08556 | 0.04514 |  |  |  |  |  |  |
| 12 |  |  | 0.05030 | 0.04183 |  |  |  |  |  |  |

+10D, 2h treatment; BMP7

|  | Rec 15min | | Rec 2h | | Rec 24h | | Rec 48h | | Rec 96h | |
| --- | --- | --- | --- | --- | --- | --- | --- | --- | --- | --- |
| Sample# | Treated | Control | Treated | Control | Treated | Control | Treated | Control | Treated | Control |
| 1 | 4.47511 | 2.65731 | 4.28756 | 3.54513 | 3.46550 | 3.13072 | 3.78895 | 2.78291 | 1.89239 | 3.43958 |
| 2 | 6.67408 | 2.21141 | 4.00796 | 5.6054 | 1.96270 | 1.54088 | 2.91814 | 3.44992 | 2.84731 | 1.02507 |
| 3 | 8.13397 | 6.49220 | 4.14151 | 4.80533 | 3.72819 | 1.56247 | 4.15939 | 3.52006 | 2.26648 | 2.22502 |
| 4 | 7.16789 | 2.04271 | 4.45858 | 4.09823 | 1.33684 | 1.07553 | 2.32281 | 2.91117 | 3.13769 | 6.00217 |
| 5 | 6.91575 | 6.06477 | 5.73534 | 0.71760 | 3.29775 | 2.58443 | 4.78936 | 3.17673 | 2.60485 | 3.43217 |
| 6 | 6.03323 | 4.19280 | 3.31062 | 6.96784 | 4.03349 | 3.71963 | 3.72047 | 3.42349 | 2.98538 | 3.19343 |
| 7 | 4.66165 | 4.41772 | 6.22537 | 5.75070 | 16.31552 | 17.40095 | 2.96147 | 1.62717 | 3.23244 | 2.58615 |
| 8 | 1.32205 | 1.23228 | 2.98217 | 3.63148 | 2.20053 | 4.01058 | 2.83348 | 4.53795 | 2.61382 | 2.60933 |
| 9 |  |  | 3.63456 | 5.02751 | 2.33419 | 2.23398 | 4.02459 | 4.56799 | 3.53971 | 2.49496 |
| 10 |  |  |  |  | 1.43496 | 2.53871 | 2.89269 | 0.92837 | 1.43716 | 4.43885 |
| 11 |  |  |  |  | 4.92197 | 3.09438 |  |  |  |  |
| 12 |  |  |  |  | 2.38172 | 1.34849 |  |  |  |  |
| 13 |  |  |  |  | 4.69467 | 4.23970 |  |  |  |  |
| 14 |  |  |  |  | 3.49756 | 3.46023 |  |  |  |  |
| 15 |  |  |  |  | 3.18449 | 0.48271 |  |  |  |  |
| 16 |  |  |  |  | 4.59751 | 3.52220 |  |  |  |  |
| 17 |  |  |  |  | 0.14968 | 5.61329 |  |  |  |  |

+10D, 48h treatment; BMP7

|  | Rec 15min | | Rec 2h | | Rec 24h | | Rec 48h | | Rec 96h | |
| --- | --- | --- | --- | --- | --- | --- | --- | --- | --- | --- |
| Sample# | Treated | Control | Treated | Control | Treated | Control | Treated | Control | Treated | Control |
| 1 | 3.78081 | 3.08394 | 4.78991 | 4.27107 | 2.09368 | 3.02134 | 3.44838 | 2.51997 | 4.97327 | 1.07537 |
| 2 | 2.41464 | 1.76259 | 1.57702 | 4.67442 | 2.33169 | 1.91800 | 2.24491 | 1.51997 | 1.73164 | 2.06683 |
| 3 | 2.49277 | 2.78297 | 5.77113 | 2.02359 | 4.75646 | 3.29823 | 1.79947 | 2.65804 | 2.59590 | 1.85675 |
| 4 | 4.34301 | 4.46344 | 4.18211 | 2.42456 | 1.29249 | 1.92327 | 2.17024 | 1.55683 | 3.18854 | 2.26219 |
| 5 | 4.82446 | 3.52882 | 4.18626 | 1.42898 | 1.77224 | 5.66757 | 3.22799 | 4.16109 | 2.71997 | 3.60901 |
| 6 | 7.30143 | 4.98261 | 2.68822 | 2.37643 | 3.18904 | 1.49735 | 4.02277 | 4.35526 | 5.57383 | 4.71103 |
| 7 | 4.92420 | 2.45872 | 1.69670 | 0.79627 | 3.54095 | 3.11881 | 2.72955 | 3.31246 | 3.33817 | 4.54965 |
| 8 | 4.37446 | 2.25037 | 1.11434 | 0.25619 | 3.28479 | 2.53291 | 2.30160 | 3.06848 | 3.56659 | 4.17137 |
| 9 |  |  | 1.77258 | 0.98745 | 3.46660 | 2.94690 |  |  | 2.87140 | 3.56229 |
| 10 |  |  | 3.47316 | 3.13680 |  |  |  |  |  |  |
| 11 |  |  | 4.65208 | 2.83553 |  |  |  |  |  |  |
| 12 |  |  | 2.59907 | 1.69196 |  |  |  |  |  |  |

1. **BMP gene expression in chick RPE over a recovery period of up to 96 h after 2 or 48 h monocular -10 D lens wear**

-10D, 2h treatment; BMP2

|  | Rec 15min | | Rec 2h | | Rec 24h | | Rec 48h | | Rec 96h | |
| --- | --- | --- | --- | --- | --- | --- | --- | --- | --- | --- |
| Sample# | Treated | Control | Treated | Control | Treated | Control | Treated | Control | Treated | Control |
| 1 | 0.07720 | 0.27010 | 1.62520 | 0.40743 | 0.93271 | 1.26205 | 0.17302 | 0.30288 | 0.17362 | 0.43095 |
| 2 | 0.10057 | 0.34436 | 1.04040 | 0.45889 | 0.24935 | 0.34199 | 0.48994 | 0.57272 | 0.39517 | 0.27419 |
| 3 | 0.06028 | 0.11300 | 0.68006 | 0.74964 | 0.44373 | 0.18718 | 0.40270 | 0.41728 | 0.18262 | 0.13280 |
| 4 | 0.08689 | 0.42831 | 1.78304 | 1.00552 | 0.82905 | 0.67735 | 0.23216 | 0.31196 | 0.21789 | 0.30531 |
| 5 | 0.04825 | 0.27416 | 1.47014 | 0.57817 | 0.23694 | 0.58087 | 0.20771 | 0.34984 | 0.96696 | 0.66241 |
| 6 | 0.07228 | 0.13847 | 1.59602 | 0.50404 | 1.03645 | 0.75398 | 0.46702 | 1.28405 | 0.14768 | 0.66830 |
| 7 | 0.11406 | 0.64746 | 2.35492 | 1.06871 | 0.21962 | 0.14528 | 0.37965 | 0.28638 | 0.77039 | 0.44455 |
| 8 | 0.14433 | 0.26375 | 1.79699 | 0.16753 | 0.16206 | 0.31433 | 0.60189 | 2.35923 | 0.51367 | 0.91043 |
| 9 | 0.05848 | 0.10516 |  |  | 0.27743 | 0.27480 | 0.88167 | 0.40368 | 0.72075 | 0.72845 |
| 10 | 0.08491 | 0.18860 |  |  | 0.44216 | 0.08983 | 0.34382 | 0.29403 | 1.81610 | 1.12806 |
| 11 |  |  |  |  |  |  | 0.24014 | 1.21425 | 1.86716 | 2.91663 |

-10D, 48h treatment; BMP2

|  | Rec 15min | | Rec 2h | | Rec 24h | | Rec 48h | | Rec 96h | |
| --- | --- | --- | --- | --- | --- | --- | --- | --- | --- | --- |
| Sample# | Treated | Control | Treated | Control | Treated | Control | Treated | Control | Treated | Control |
| 1 | 0.26514 | 0.58647 | 0.70088 | 0.38694 | 0.11038 | 0.09628 | 0.62132 | 0.28373 | 0.80990 | 0.84280 |
| 2 | 0.80348 | 1.31420 | 0.10751 | 0.54514 | 0.63117 | 0.23374 | 0.47657 | 0.21445 | 0.49724 | 0.39448 |
| 3 | 0.15587 | 0.51537 | 0.67770 | 0.02235 | 0.98225 | 0.58237 | 1.32825 | 1.09758 | 0.28339 | 1.52844 |
| 4 | 0.09239 | 0.83387 | 1.35244 | 0.10988 | 0.74306 | 0.80615 | 0.70513 | 0.53587 | 0.71106 | 0.94817 |
| 5 | 0.23309 | 1.25779 | 1.40224 | 0.15538 | 0.59456 | 0.31267 | 0.84567 | 0.50520 | 0.34084 | 0.36458 |
| 6 | 0.30466 | 0.56219 | 2.12406 | 0.23745 | 0.33126 | 0.19951 | 1.25665 | 1.02383 | 0.53407 | 0.24927 |
| 7 | 0.34066 | 1.08039 | 1.08658 | 0.28487 | 1.95456 | 0.20723 | 0.37076 | 0.41075 | 0.75381 | 0.31062 |
| 8 | 0.38350 | 2.06774 | 1.67593 | 0.75192 | 1.10283 | 0.18663 | 1.01961 | 0.48716 | 0.23305 | 0.22982 |
| 9 | 0.29059 | 0.96063 | 0.80781 | 0.19028 | 0.84946 | 0.26814 | 1.50664 | 0.62496 | 0.55795 | 0.01780 |
| 10 |  |  | 0.39257 | 0.11665 | 1.01651 | 0.37947 | 0.35299 | 0.53784 | 0.26162 | 0.29322 |
| 11 |  |  | 0.78007 | 0.09591 |  |  | 0.53708 | 0.61115 | 0.09150 | 0.43089 |
| 12 |  |  | 0.81959 | 0.27352 |  |  | 0.37543 | 0.37746 |  |  |
| 13 |  |  |  |  |  |  | 0.52367 | 0.06148 |  |  |

-10D, 2h treatment; BMP4

|  | Rec 15min | | Rec 2h | | Rec 24h | | Rec 48h | | Rec 96h | |
| --- | --- | --- | --- | --- | --- | --- | --- | --- | --- | --- |
| Sample# | Treated | Control | Treated | Control | Treated | Control | Treated | Control | Treated | Control |
| 1 | 0.08570 | 0.10676 | 0.19801 | 0.14050 | 0.43926 | 0.49087 | 0.13847 | 0.16814 | 0.30330 | 0.54749 |
| 2 | 0.06635 | 0.10846 | 0.25498 | 0.22634 | 0.28822 | 0.34366 | 0.20656 | 0.30391 | 0.27190 | 0.28872 |
| 3 | 0.05769 | 0.05476 | 0.39609 | 0.36649 | 0.21731 | 0.09965 | 0.25333 | 0.28997 | 0.11517 | 0.09186 |
| 4 | 0.07877 | 0.13671 | 0.51362 | 0.38083 | 0.23053 | 0.11515 | 0.09881 | 0.12938 | 0.17007 | 0.31468 |
| 5 | 0.03060 | 0.07855 | 0.61120 | 0.40972 | 0.09436 | 0.21027 | 0.09561 | 0.16121 | 0.44507 | 0.38291 |
| 6 | 0.04749 | 0.05137 | 0.92835 | 0.50847 | 0.25623 | 0.23997 | 0.81348 | 0.50005 | 0.13661 | 0.59952 |
| 7 | 0.09765 | 0.25955 | 1.01625 | 0.70483 | 0.19645 | 0.18570 | 0.26081 | 0.25371 | 0.67331 | 0.43802 |
| 8 | 0.10748 | 0.11480 | 0.24168 | 0.09512 | 0.17432 | 0.16867 | 0.37770 | 0.99436 | 0.51049 | 0.76118 |
| 9 | 0.03586 | 0.05763 |  |  | 0.20999 | 0.20823 | 0.23911 | 0.13651 | 0.56575 | 0.39543 |
| 10 | 0.05356 | 0.05154 |  |  | 0.27107 | 0.08240 | 0.23747 | 0.20553 | 0.70722 | 0.53385 |
| 11 |  |  |  |  |  |  | 0.07889 | 0.40367 | 0.70634 | 0.74228 |

-10D, 48h treatment; BMP4

|  | Rec 15min | | Rec 2h | | Rec 24h | | Rec 48h | | Rec 96h | |
| --- | --- | --- | --- | --- | --- | --- | --- | --- | --- | --- |
| Sample# | Treated | Control | Treated | Control | Treated | Control | Treated | Control | Treated | Control |
| 1 | 0.29808 | 0.32028 | 0.16492 | 0.18291 | 0.23785 | 0.20537 | 0.14889 | 0.11622 | 0.30098 | 0.42496 |
| 2 | 0.34339 | 0.63694 | 0.05417 | 0.36911 | 0.24360 | 0.30459 | 0.19229 | 0.10178 | 0.28058 | 0.22954 |
| 3 | 0.14795 | 0.28469 | 0.26718 | 0.02120 | 0.40038 | 0.36437 | 0.30778 | 0.32768 | 0.28698 | 0.58194 |
| 4 | 0.18437 | 0.44233 | 0.30178 | 0.09904 | 0.29905 | 0.36081 | 0.29004 | 0.26861 | 0.40394 | 0.49581 |
| 5 | 0.12941 | 0.64601 | 0.40229 | 0.10720 | 0.30215 | 0.18607 | 0.38529 | 0.25780 | 0.37721 | 0.30869 |
| 6 | 0.22243 | 0.28049 | 0.25090 | 0.13210 | 0.16303 | 0.20455 | 0.47938 | 0.39446 | 0.34786 | 0.20477 |
| 7 | 0.26751 | 0.58884 | 0.25153 | 0.11947 | 0.64761 | 0.21005 | 0.16471 | 0.19377 | 0.47551 | 0.31185 |
| 8 | 0.32675 | 0.65884 | 0.48942 | 0.41264 | 0.42108 | 0.10325 | 0.38113 | 0.19906 | 0.21045 | 0.24002 |
| 9 | 0.26963 | 0.54209 | 0.45883 | 0.18158 | 0.42433 | 0.24358 | 0.73428 | 0.40917 | 0.39100 | 0.02324 |
| 10 |  |  | 0.14722 | 0.18141 | 0.57102 | 0.29200 | 0.31502 | 0.27147 | 0.41650 | 0.51227 |
| 11 |  |  | 0.36629 | 0.14387 |  |  | 0.41321 | 0.41636 | 0.11651 | 0.21256 |
| 12 |  |  | 0.31137 | 0.15425 |  |  | 0.18987 | 0.17302 |  |  |
| 13 |  |  |  |  |  |  | 0.23794 | 0.03369 |  |  |

-10D, 2h treatment; BMP7

|  | Rec 15min | | Rec 2h | | Rec 24h | | Rec 48h | | Rec 96h | |
| --- | --- | --- | --- | --- | --- | --- | --- | --- | --- | --- |
| Sample# | Treated | Control | Treated | Control | Treated | Control | Treated | Control | Treated | Control |
| 1 | 3.54820 | 3.44852 | 1.95747 | 2.08709 | 2.73470 | 4.25670 | 2.45637 | 2.37005 | 2.66417 | 2.98109 |
| 2 | 3.68518 | 4.34792 | 2.20494 | 3.76378 | 3.14701 | 2.85544 | 2.53191 | 3.25459 | 2.69272 | 4.12437 |
| 3 | 2.26081 | 1.52715 | 3.18833 | 4.56582 | 2.78084 | 1.80704 | 3.32920 | 3.82546 | 1.26757 | 0.99786 |
| 4 | 3.83177 | 5.02564 | 5.21249 | 1.16906 | 4.53644 | 2.66036 | 2.12573 | 2.35368 | 1.56937 | 2.96809 |
| 5 | 2.03272 | 2.34051 | 5.04053 | 2.25896 | 1.02022 | 2.09303 | 1.17530 | 1.83827 | 4.98522 | 3.97976 |
| 6 | 2.84574 | 2.46306 | 6.05753 | 2.84177 | 2.72572 | 2.59336 | 2.96332 | 2.62697 | 0.75572 | 2.50751 |
| 7 | 3.87553 | 6.50204 | 6.90651 | 6.26665 | 3.00540 | 3.59852 | 3.09904 | 2.62412 | 2.75163 | 1.50541 |
| 8 | 5.61417 | 2.79219 | 6.67885 | 4.47648 | 2.73133 | 2.41043 | 4.02315 | 4.69916 | 3.56304 | 3.86736 |
| 9 | 1.80472 | 2.44030 |  |  | 3.65455 | 3.84289 | 4.94221 | 3.23995 | 3.86381 | 2.67200 |
| 10 | 4.75019 | 3.52947 |  |  | 3.11852 | 1.24125 | 3.47714 | 4.28625 | 4.64827 | 4.39311 |
| 11 |  |  |  |  |  |  | 2.94490 | 5.13332 | 4.92967 | 4.59274 |

-10D, 48h treatment; BMP7

|  | Rec 15min | | Rec 2h | | Rec 24h | | Rec 48h | | Rec 96h | |
| --- | --- | --- | --- | --- | --- | --- | --- | --- | --- | --- |
| Sample# | Treated | Control | Treated | Control | Treated | Control | Treated | Control | Treated | Control |
| 1 | 7.18865 | 6.31517 | 2.60116 | 2.80818 | 3.41169 | 3.55498 | 4.10832 | 3.14080 | 9.79384 | 6.77728 |
| 2 | 4.45371 | 5.67149 | 0.69357 | 5.49837 | 4.57305 | 4.62023 | 4.56007 | 2.08767 | 4.14392 | 2.90820 |
| 3 | 3.87074 | 6.05011 | 4.43737 | 0.62524 | 5.98998 | 5.20785 | 2.98635 | 3.33588 | 3.59668 | 8.41867 |
| 4 | 4.39254 | 6.31589 | 3.39180 | 2.46488 | 4.48892 | 4.31591 | 2.05628 | 3.24162 | 4.56164 | 4.78311 |
| 5 | 1.88856 | 5.71737 | 5.68140 | 2.68803 | 4.85052 | 3.90178 | 2.78857 | 2.50664 | 6.05766 | 4.97347 |
| 6 | 5.17963 | 4.12824 | 7.39832 | 8.44518 | 3.05556 | 3.17186 | 4.49552 | 4.81723 | 7.32839 | 4.27332 |
| 7 | 4.69549 | 5.84320 | 6.28140 | 3.46697 | 7.17011 | 6.09027 | 3.18674 | 3.96743 | 7.62297 | 6.06711 |
| 8 | 7.50153 | 8.24607 | 6.91469 | 7.17211 | 4.43377 | 3.04178 | 7.49563 | 4.95080 | 3.38130 | 3.86963 |
| 9 | 7.27975 | 6.59743 | 5.44575 | 4.01949 | 6.59196 | 5.02878 | 5.10062 | 4.86122 | 4.60925 | 0.39252 |
| 10 |  |  | 1.70001 | 4.42245 | 6.77430 | 4.36198 | 5.62899 | 3.82265 | 4.31321 | 4.83136 |
| 11 |  |  | 5.66505 | 5.63181 |  |  | 3.76719 | 4.42043 | 3.29086 | 4.87506 |
| 12 |  |  | 7.69887 | 4.01170 |  |  | 5.11073 | 4.72249 |  |  |
| 13 |  |  |  |  |  |  | 6.07237 | 0.65444 |  |  |

1. **BMP gene expression in chick RPE over a recovery period of up to 96 h after 2 or 48 h monocular FD treatment**

FD, 2h treatment; BMP2

|  | Rec 15min | | Rec 2h | | Rec 24h | | Rec 48h | | Rec 96h | |
| --- | --- | --- | --- | --- | --- | --- | --- | --- | --- | --- |
| Sample# | Treated | Control | Treated | Control | Treated | Control | Treated | Control | Treated | Control |
| 1 | 0.06107 | 0.02510 | 0.21776 | 0.11579 | 0.21750 | 0.40055 | 0.61015 | 0.72027 | 0.10644 | 0.41215 |
| 2 | 0.06823 | 0.15864 | 1.36497 | 0.60140 | 0.75055 | 0.32647 | 0.04846 | 0.76707 | 0.36427 | 0.22512 |
| 3 | 0.06156 | 0.37422 | 0.57318 | 0.36647 | 0.07575 | 0.02369 | 0.57266 | 0.89911 | 0.19353 | 0.24672 |
| 4 | 0.09781 | 0.55370 | 1.35756 | 0.50800 | 0.74086 | 0.17391 | 0.64527 | 1.05231 | 0.30340 | 0.42327 |
| 5 | 0.21623 | 0.60050 | 0.61703 | 0.27857 | 0.15777 | 0.35620 | 0.58469 | 0.31256 | 0.62564 | 0.43344 |
| 6 | 0.03379 | 0.35915 | 1.50399 | 0.81119 | 0.07741 | 0.99917 | 0.55861 | 0.20933 | 0.06672 | 0.28586 |
| 7 | 0.06189 | 0.25915 | 1.41748 | 0.14052 | 0.06460 | 0.22827 | 1.66640 | 0.37346 | 1.00459 | 0.54871 |
| 8 | 0.05851 | 0.55680 | 1.44147 | 0.35031 | 0.45230 | 0.41857 | 0.10475 | 0.21644 | 0.65865 | 0.88066 |
| 9 | 0.06159 | 0.23537 | 0.56047 | 0.20703 | 0.49709 | 0.31011 |  |  |  |  |
| 10 |  |  | 1.52269 | 0.71084 | 0.84819 | 0.42684 |  |  |  |  |
| 11 |  |  |  |  | 0.06942 | 0.53958 |  |  |  |  |
| 12 |  |  |  |  | 0.76962 | 0.42754 |  |  |  |  |

FD, 48h treatment; BMP2

|  | Rec 15min | | Rec 2h | | Rec 24h | | Rec 48h | | Rec 96h | |
| --- | --- | --- | --- | --- | --- | --- | --- | --- | --- | --- |
| Sample# | Treated | Control | Treated | Control | Treated | Control | Treated | Control | Treated | Control |
| 1 | 0.13732 | 0.77963 | 0.87106 | 0.26783 | 0.70826 | 1.04531 | 0.82649 | 0.33903 | 0.06963 | 0.64085 |
| 2 | 0.19677 | 0.79695 | 0.92039 | 0.57013 | 2.84296 | 1.26596 | 0.27123 | 0.18254 | 0.58619 | 0.12288 |
| 3 | 0.10112 | 0.45852 | 1.67233 | 0.58917 | 0.13011 | 0.18221 | 0.64605 | 0.38793 | 0.18211 | 0.17234 |
| 4 | 0.03522 | 0.84424 | 2.45441 | 0.45856 | 0.77694 | 0.35618 | 0.54827 | 0.48650 | 0.45258 | 7.04087 |
| 5 | 0.15440 | 0.39578 | 1.46164 | 0.59275 | 2.60114 | 0.19303 | 0.37481 | 0.33069 | 0.26490 | 0.73671 |
| 6 | 0.10874 | 0.12135 | 1.03576 | 0.28992 | 1.12528 | 0.34098 | 0.94509 | 0.55124 | 0.23902 | 0.37021 |
| 7 | 0.05123 | 0.51073 | 1.74671 | 0.14313 | 1.72473 | 0.34556 | 0.26675 | 0.34247 | 0.42621 | 0.17046 |
| 8 | 0.08417 | 0.26866 | 1.04795 | 0.06403 | 0.51369 | 0.46955 | 0.55132 | 0.60350 | 0.32036 | 0.51642 |
| 9 | 0.08323 | 0.32805 | 1.43652 | 1.24475 | 0.33937 | 0.13081 | 0.67584 | 0.40604 | 0.66702 | 0.39053 |
| 10 | 0.08022 | 0.16346 |  |  | 0.20984 | 0.08858 | 1.00873 | 0.71628 | 0.29291 | 0.55896 |
| 11 | 0.04829 | 0.28572 |  |  | 0.50985 | 0.44302 | 0.68152 | 1.17060 |  |  |
| 12 |  |  |  |  | 0.27267 | 0.38858 | 2.01607 | 1.17273 |  |  |

FD, 2h treatment; BMP4

|  | Rec 15min | | Rec 2h | | Rec 24h | | Rec 48h | | Rec 96h | |
| --- | --- | --- | --- | --- | --- | --- | --- | --- | --- | --- |
| Sample# | Treated | Control | Treated | Control | Treated | Control | Treated | Control | Treated | Control |
| 1 | 0.08029 | 0.00911 | 0.07711 | 0.05841 | 0.08826 | 0.10657 | 0.18623 | 0.19535 | 0.05130 | 0.11631 |
| 2 | 0.11861 | 0.11495 | 0.22412 | 0.14608 | 0.26535 | 0.13536 | 0.01403 | 0.16772 | 0.15111 | 0.10739 |
| 3 | 0.10047 | 0.11357 | 0.15738 | 0.08440 | 0.02508 | 0.01050 | 0.13324 | 0.14323 | 0.14660 | 0.18117 |
| 4 | 0.11434 | 0.18827 | 0.12314 | 0.13357 | 0.28004 | 0.10995 | 0.19281 | 0.23230 | 0.13285 | 0.17349 |
| 5 | 0.18569 | 0.37187 | 0.10003 | 0.04177 | 0.06545 | 0.17417 | 0.17204 | 0.12576 | 0.39587 | 0.35743 |
| 6 | 0.03537 | 0.22882 | 0.27439 | 0.10461 | 0.03044 | 0.27037 | 0.22390 | 0.15570 | 0.21826 | 0.31327 |
| 7 | 0.05622 | 0.12951 | 0.10722 | 0.02578 | 0.01795 | 0.04634 | 0.47586 | 0.24125 | 0.45589 | 0.16766 |
| 8 | 0.05949 | 0.17728 | 0.42803 | 0.16740 | 0.20261 | 0.16232 | 0.03194 | 0.10522 | 0.21099 | 0.22168 |
| 9 | 0.07175 | 0.16772 | 0.15094 | 0.07036 | 0.07724 | 0.07458 |  |  |  |  |
| 10 |  |  | 0.28901 | 0.15708 | 0.22568 | 0.15879 |  |  |  |  |
| 11 |  |  |  |  | 0.02340 | 0.12311 |  |  |  |  |
| 12 |  |  |  |  | 0.12175 | 0.10943 |  |  |  |  |

FD, 48h treatment; BMP4

|  | Rec 15min | | Rec 2h | | Rec 24h | | Rec 48h | | Rec 96h | |
| --- | --- | --- | --- | --- | --- | --- | --- | --- | --- | --- |
| Sample# | Treated | Control | Treated | Control | Treated | Control | Treated | Control | Treated | Control |
| 1 | 0.08758 | 0.19500 | 0.12011 | 0.07217 | 0.14249 | 0.14782 | 0.13989 | 0.06935 | 0.01872 | 0.14394 |
| 2 | 0.09992 | 0.20462 | 0.16813 | 0.16982 | 0.65694 | 0.36843 | 0.18419 | 0.11932 | 0.22796 | 0.03931 |
| 3 | 0.04207 | 0.09341 | 0.15625 | 0.10417 | 0.11201 | 0.16390 | 0.17859 | 0.14528 | 0.13193 | 0.07362 |
| 4 | 0.01256 | 0.14828 | 0.21066 | 0.08238 | 0.27378 | 0.15692 | 0.11299 | 0.09126 | 0.17694 | 0.18946 |
| 5 | 0.09101 | 0.06635 | 0.21843 | 0.13156 | 0.56762 | 0.09618 | 0.10342 | 0.13449 | 0.19845 | 0.44971 |
| 6 | 0.10974 | 0.05572 | 0.13773 | 0.08206 | 0.38724 | 0.19470 | 0.27563 | 0.15577 | 0.10999 | 0.15099 |
| 7 | 0.09385 | 0.14200 | 0.16784 | 0.05759 | 0.23662 | 0.08646 | 0.11961 | 0.13224 | 0.12013 | 0.04752 |
| 8 | 0.11755 | 0.13604 | 0.11659 | 0.00859 | 0.15435 | 0.17371 | 0.14811 | 0.15332 | 0.14471 | 0.20489 |
| 9 | 0.08958 | 0.08069 | 0.08886 | 0.15243 | 0.05036 | 0.03526 | 0.22315 | 0.15387 | 0.14507 | 0.09731 |
| 10 | 0.08132 | 0.10706 |  |  | 0.03621 | 0.03269 | 0.33169 | 0.22342 | 0.05591 | 0.14392 |
| 11 | 0.04851 | 0.08121 |  |  | 0.13934 | 0.07928 | 0.16871 | 0.22291 |  |  |
| 12 |  |  |  |  | 0.08494 | 0.06440 | 0.33490 | 0.20915 |  |  |

FD, 2h treatment; BMP7

|  | Rec 15min | | Rec 2h | | Rec 24h | | Rec 48h | | Rec 96h | |
| --- | --- | --- | --- | --- | --- | --- | --- | --- | --- | --- |
| Sample# | Treated | Control | Treated | Control | Treated | Control | Treated | Control | Treated | Control |
| 1 | 2.37112 | 0.28410 | 1.26374 | 1.62898 | 1.45424 | 2.07293 | 3.10911 | 3.61853 | 1.30241 | 2.92755 |
| 2 | 3.12675 | 3.20326 | 3.23926 | 3.21312 | 3.20361 | 2.55632 | 0.34260 | 4.47618 | 2.63176 | 2.24989 |
| 3 | 3.05631 | 4.31116 | 3.99006 | 3.36471 | 0.12965 | 0.10006 | 5.00144 | 5.13233 | 3.32148 | 3.74311 |
| 4 | 3.22331 | 3.67965 | 3.50655 | 5.19124 | 3.14409 | 2.58901 | 5.02287 | 4.96599 | 3.01976 | 3.27436 |
| 5 | 3.83125 | 4.01618 | 2.22782 | 1.20890 | 2.24735 | 3.87207 | 3.53815 | 3.46617 | 5.24302 | 3.49700 |
| 6 | 1.04631 | 6.01346 | 2.94895 | 1.66509 | 0.55103 | 4.33672 | 2.94411 | 2.85728 | 1.70953 | 2.67538 |
| 7 | 1.90644 | 2.82549 | 3.59491 | 0.94975 | 1.03615 | 1.85952 | 4.23654 | 2.85313 | 4.96392 | 2.20020 |
| 8 | 4.17956 | 4.72997 | 5.37563 | 2.93484 | 3.16455 | 3.74652 | 0.71848 | 2.20051 | 2.79908 | 2.95468 |
| 9 | 2.79199 | 4.22008 | 3.50379 | 2.31379 | 2.78405 | 2.54353 |  |  |  |  |
| 10 |  |  | 3.55491 | 2.88274 | 3.24284 | 3.90119 |  |  |  |  |
| 11 |  |  |  |  | 0.79177 | 3.89690 |  |  |  |  |
| 12 |  |  |  |  | 6.34368 | 6.11548 |  |  |  |  |

FD, 48h treatment; BMP7

|  | Rec 15min | | Rec 2h | | Rec 24h | | Rec 48h | | Rec 96h | |
| --- | --- | --- | --- | --- | --- | --- | --- | --- | --- | --- |
| Sample# | Treated | Control | Treated | Control | Treated | Control | Treated | Control | Treated | Control |
| 1 | 3.41092 | 5.32113 | 3.27843 | 2.97993 | 2.98919 | 3.41286 | 4.83281 | 4.43306 | 0.71490 | 4.33543 |
| 2 | 3.01816 | 4.07086 | 2.55533 | 3.22131 | 1.74582 | 0.73910 | 6.44953 | 4.61568 | 5.60916 | 0.91236 |
| 3 | 1.81577 | 3.18165 | 3.32219 | 3.38405 | 2.51180 | 3.48658 | 4.24021 | 5.66956 | 4.36997 | 2.26518 |
| 4 | 0.42037 | 3.94133 | 5.15374 | 3.78521 | 3.07040 | 2.74229 | 4.05727 | 4.35667 | 3.10566 | 3.21635 |
| 5 | 2.88100 | 1.56051 | 2.96563 | 3.38997 | 6.78948 | 2.36154 | 3.35485 | 3.90515 | 2.96408 | 4.09270 |
| 6 | 2.73143 | 1.87950 | 3.36800 | 2.31707 | 6.56643 | 3.44278 | 7.35659 | 4.80216 | 3.23653 | 3.55462 |
| 7 | 2.23485 | 3.98392 | 1.94482 | 2.17645 | 3.50581 | 2.89271 | 3.94285 | 4.85341 | 2.66457 | 1.00527 |
| 8 | 3.59160 | 4.43805 | 3.91552 | 0.37548 | 2.79096 | 3.47803 | 3.29737 | 4.07179 | 3.40823 | 4.83702 |
| 9 | 3.32507 | 3.53296 | 3.92633 | 6.77555 | 1.30494 | 1.26766 | 4.79739 | 3.56509 | 4.47222 | 4.28742 |
| 10 | 2.81503 | 3.31303 |  |  | 0.86806 | 1.16527 | 7.45991 | 4.90305 | 1.44085 | 4.13389 |
| 11 | 2.11345 | 3.25987 |  |  | 4.67134 | 5.19417 | 3.96270 | 5.34902 |  |  |
| 12 |  |  |  |  | 3.58780 | 2.48344 | 5.61767 | 5.23295 |  |  |
